# EspM Is a Conserved Transcription Factor That Regulates Gene Expression in Response to the ESX-1 System

**DOI:** 10.1128/mBio.02807-19

**Published:** 2020-02-04

**Authors:** Kevin G. Sanchez, Micah J. Ferrell, Alexandra E. Chirakos, Kathleen R. Nicholson, Robert B. Abramovitch, Matthew M. Champion, Patricia A. Champion

**Affiliations:** a Department of Biological Sciences, University of Notre Dame, Notre Dame, Indiana, USA; b Department of Microbiology and Molecular Genetics, Michigan State University, East Lansing, Michigan, USA; c Department of Chemistry and Biochemistry, University of Notre Dame, Notre Dame, Indiana, USA; Washington University School of Medicine in St. Louis

**Keywords:** ESAT-6, ESX-1, *Mycobacterium*, protein secretion, regulation, feedback control

## Abstract

Pathogenic mycobacteria encounter multiple environments during macrophage infection. Temporally, the bacteria are engulfed into the phagosome, lyse the phagosomal membrane, and interact with the cytosol before spreading to another cell. Virulence factors secreted by the mycobacterial ESX-1 (ESAT-6-system-1) secretion system mediate the essential transition from the phagosome to the cytosol. It was recently discovered that the ESX-1 system also regulates mycobacterial gene expression in Mycobacterium marinum (R. E. Bosserman, T. T. Nguyen, K. G. Sanchez, A. E. Chirakos, et al., Proc Natl Acad Sci U S A 114:E10772–E10781, 2017, https://doi.org/10.1073/pnas.1710167114), a nontuberculous mycobacterial pathogen, and in the human-pathogenic species M. tuberculosis (A. M. Abdallah, E. M. Weerdenburg, Q. Guan, R. Ummels, et al., PLoS One 14:e0211003, 2019, https://doi.org/10.1371/journal.pone.0211003). It is not known how the ESX-1 system regulates gene expression. Here, we identify the first transcription factor required for the ESX-1-dependent transcriptional response in pathogenic mycobacteria. We demonstrate that the gene divergently transcribed from the *whiB6* gene and adjacent to the ESX-1 locus in mycobacterial pathogens encodes a conserved transcription factor (*MMAR_5438*, *Rv3863*, now *espM*). We prove that EspM from both M. marinum and M. tuberculosis directly and specifically binds the *whiB6-espM* intergenic region. We show that EspM is required for ESX-1-dependent repression of *whiB6* expression and for the regulation of *ESX-1*-associated gene expression. Finally, we demonstrate that EspM functions to fine-tune ESX-1 activity in M. marinum. Taking the data together, this report extends the *esx-1* locus, defines a conserved regulator of the ESX-1 virulence pathway, and begins to elucidate how the ESX-1 system regulates gene expression.

## Abstract (precis)

Mycobacterial pathogens use the ESX-1 system to transport protein substrates that mediate essential interactions with the host during infection. We previously demonstrated that in addition to transporting proteins, the ESX-1 secretion system regulates gene expression. Here, we identify a conserved transcription factor that regulates gene expression in response to the ESX-1 system. We demonstrate that this transcription factor is functionally conserved in M. marinum, a pathogen of ectothermic animals; M. tuberculosis, the human-pathogenic species that causes tuberculosis; and M. smegmatis, a nonpathogenic mycobacterial species. These findings provide the first mechanistic insight into how the ESX-1 system elicits a transcriptional response, a function of this protein transport system that was previously unknown.

## INTRODUCTION

Following infection, pathogenic mycobacteria, including Mycobacterium tuberculosis, are engulfed by macrophages and reside in the phagosome (1–3). Survival in the phagosome requires regulated changes in bacterial gene expression (1, 4). Pathogenic mycobacteria use the ESX-1 secretion system (SS) to lyse the phagosome and mediate bacterial access the cytoplasm (5–13). The ESX-1 system is functionally conserved between M. tuberculosis, the cause of human tuberculosis, and Mycobacterium marinum, a pathogen of poikilothermic fish and an established model for the ESX-1 system (14–18). Phagosomal lysis releases secreted bacterial factors and triggers the host response to infection (7, 8, 19–27). In the absence of an ESX-1 system, both mycobacterial pathogens remain in the phagosome and are attenuated (7–9, 22).

Several ESX-1 conserved components (Ecc’s) form a complex in the cytoplasmic membrane (CM). The ESX-1 membrane complex recognizes ESX-1 substrates and provides the energy and the pore for the export of ESX-1 substrates across the CM (28, 29). The protein substrates are then translocated across the periplasm and mycolate outer membrane via an unknown process (30). ESX-1 substrates can be localized to the cell surface and/or secreted from the bacterial cell into the extracellular environment (31–34). We recently demonstrated that, in addition to transporting proteins, the presence or absence of the ESX-1 membrane complex in the CM elicits a widespread transcriptional response, a previously unrecognized function of the ESX-1 system (35). ESX-1-dependent gene expression has since been confirmed in M. marinum and reported in M. tuberculosis (36, 37).

The ESX-1-dependent transcriptional response includes a negative-feedback mechanism linking the levels of ESX-1 substrates to the presence or absence of the ESX-1 membrane complex (35). WhiB6 is a stress-responsive transcription factor (38, 39) that directly activates ESX-1 substrate gene expression in M. marinum and in M. tuberculosis (38, 39). The ESX-1 system regulates *whiB6* gene expression both in M. marinum and in M. tuberculosis (35–37). In the presence of the ESX-1 membrane complex, the *whiB6* gene is expressed, and there is WhiB6-dependent expression of the genes encoding ESX-1 substrates. In the absence of the ESX-1 membrane complex, *whiB6* gene expression, as well as the expression of ESX-1 substrate genes, is significantly reduced (35, 36). How the ESX-1 membrane complex regulates *whiB6* gene expression is unknown.

On the basis of our published data and of those published previously by independent groups, ESX-1-dependent changes in gene expression cannot be explained by the loss of the WhiB6 transcription factor alone (35–37). Therefore, we hypothesized that additional transcription factors regulate genes in response to the presence of the ESX-1 membrane complex.

## RESULTS

### The EspM protein binds upstream of the *whiB6* gene.

**FIG 1:**
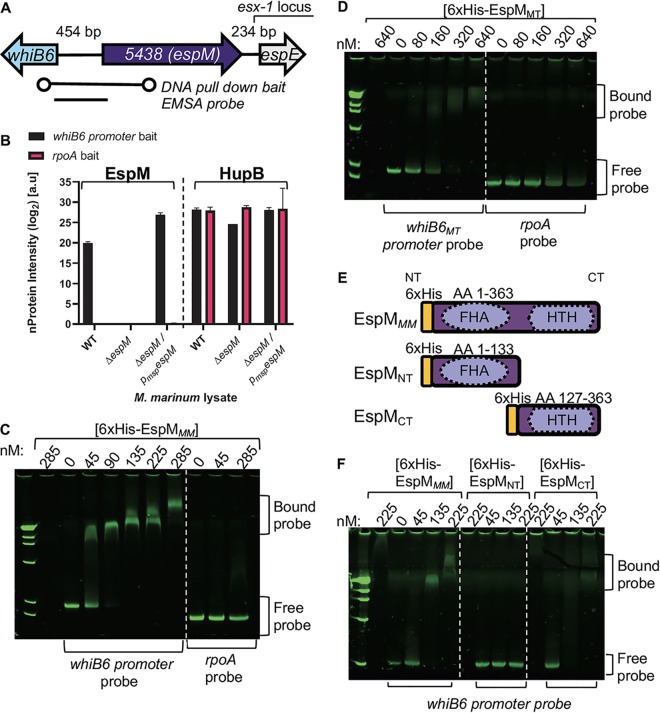
Identification of MMAR_5438 (EspM) as a DNA-binding protein in M. marinum. (A) The *whiB6* gene is separated from the *esx-1* locus by the *MMAR_5438* gene. The biotinylated 1-kb probe (circles) for the DNA pulldown is indicated. The 500-bp probe for the EMSA analysis is indicated in panel C. (B) MS analysis of the DNA pulldown showing the enrichment of the EspM and HupB proteins. The scale represents normalized MS peak area intensity levels. a.u, arbitrary units. (C and D) EMSAs performed with increasing concentrations of the 6×His-EspM protein from M. marinum (EspM*_MM_*) (C) or the 6×His-EspM protein from M. tuberculosis (EspM*_MT_*) (D). The control probe used as indicated in both panels was 500 bp of the *rpoA* open reading frame (ORF) (bp 1309999 to 1310499) from M. marinum. (E) Schematic of the 6×His-EspM*_MM_* proteins affinity purified from E. coli used in the EMSAs. (F) EMSA performed with the *whiB6-espM* probe with increasing amounts of EspM*_MM_*, EspM*_NT_*, and EspM*_CT_* from M. marinum.

To identify transcription factors that regulate genes in response to the ESX-1 membrane complex, we focused on the regulation of the *whiB6* gene. The 1 kb of DNA upstream of the *whiB6* gene is sufficient for regulation of *whiB6* gene expression by the ESX-1 membrane complex (35). We used a DNA pulldown to enrich proteins from M. marinum lysate that specifically bind the 1 kb of DNA upstream of the *whiB6* gene (“*whiB6* promoter bait,” Fig. 1A; bp 6577326 to 6578305 in the M. marinum genome). Using liquid chromatograph-tandem mass spectrometry (LC-MS/MS)-based quantitative proteomics on the proteins eluted from the DNA, we identified several proteins that were specifically and reproducibly enriched for binding the *whiB6* promoter bait relative to binding nonspecific DNA (*rpoA* bait; see Table S1 in the supplemental material). MMAR_5438 was enriched for binding the *whiB6* promoter bait ≥64.0-fold ± 0.4-fold relative to the *rpoA* bait (Fig. 1B). We propose renaming the *MMAR_5438* gene “*espM*,” consistent with current ESX-1 nomenclature (40). We generated an M. marinum strain with an unmarked deletion of the *espM* gene (Δ*espM*; Fig. S1) and a complementation strain with an integrated constitutive *espM* expression plasmid (Δ*espM*/p*_msp_espM*). The EspM protein was not identified in the DNA pulldown performed with lysate from the Δ*espM* strain and was further enriched for *whiB6* promoter bait binding compared to the *rpoA* bait in lysates from the complemented strain (Fig. 1B). We also identified several M. marinum proteins with known DNA binding activity that were not significantly or reproducibly enriched for binding the *whiB6* promoter bait relative to the *rpoA* bait (Table S1). For example, the M. marinum DNA-binding protein Hu homolog HupB (MMAR_1728) bound the two baits comparably following incubation with any M. marinum lysate (Fig. 1B).

10.1128/mBio.02807-19.2 FIG S1 Generation and confirmation of M. marinum Δ*espM* strain. (A) Schematic of parental, Δ*MMAR_5438* (Δ*espM*), and complemented strains. (B) Confirmation of Δ*espM* genotype. Diluted lysates of resolved strains were checked by PCR performed with primers OMF619 and OMF620. Download FIG S1, PDF file, 0.4 MB. Copyright © 2020 Sanchez et al. 2020 Sanchez et al. This content is distributed under the terms of the Creative Commons Attribution 4.0 International license.

10.1128/mBio.02807-19.8 TABLE S1 (A to D) Processed MS data from the DNA affinity chromatography assay performed as described in the Fig. 1 legend. (A) Unfiltered processed protein data. (B) Filtered data used for quantitation, (C) Normalized LFQ enrichment for MMAR_5438 and HupB DNA binding. (D) Proteins enriched for binding of the *whiB6-espM* probe (>2-fold enrichment over the *rpoA* probe level). (E and F) Relative quantification of the proteins described in the Fig. S4E legend. (E) Unfiltered processed protein group data. (F) Filtered data used for quantification. For panels E and F, two biological replicates each were performed in technical duplicate. The data shown are representative of results from one biological replicate, performed in technical duplicate. Download Table S1, XLSX file, 1.9 MB. Copyright © 2020 Sanchez et al. 2020 Sanchez et al. This content is distributed under the terms of the Creative Commons Attribution 4.0 International license.

To confirm the interaction of the EspM protein with the *whiB6* promoter region, we expressed and purified an N-terminally 6×His-tagged EspM*_MM_* fusion protein from Escherichia coli (the *MM* subscript refers to the protein from M. marinum [40]) (see Fig. S2 in the supplemental material) and performed electrophoretic mobility shift assays (EMSAs). We observed a specific shift in mobility of the *whiB6* promoter probe (550 bp) (Fig. 1A, “EMSA probe”) and a concomitant loss of free *whiB6* promoter probe with increasing concentrations of the 6×His-EspM*_MM_* protein (Fig. 1C). We did not observe a mobility shift of the *rpoA* probe, confirming the specific binding of the EspM*_MM_* protein to the *whiB6* promoter probe.

10.1128/mBio.02807-19.3 FIG S2 Heterologous expression of EspM proteins from M. marinum and M. tuberculosis. (A to C) Coomassie-stained gels representing purification of 6×His-EspM*_MM_* protein (A) and of denatured 6×His-EspM*_MT_* with refolding (B) and dilution series of purified 6×His-EspMNT (aa 1 to 133) and 6×His-EspMCT (aa 127 to 363) (C) from M. marinum. (D) Purification of WhiB6*_MM_*-6×His protein. Final concentrations and buffer conditions are listed in Text S1. Download FIG S2, PDF file, 1.3 MB. Copyright © 2020 Sanchez et al. 2020 Sanchez et al. This content is distributed under the terms of the Creative Commons Attribution 4.0 International license.

10.1128/mBio.02807-19.1 TEXT S1 Detailed Materials and Methods section regarding the approaches used in this article. Download Text S1, PDF file, 0.2 MB. Copyright © 2020 Sanchez et al. 2020 Sanchez et al. This content is distributed under the terms of the Creative Commons Attribution 4.0 International license.

The *espM* gene is conserved in M. tuberculosis. The EspM proteins in M. marinum and M. tuberculosis Erdman (ERDMAN_4236, EspM*_MT_*) are 76.25% identical at the amino acid level (41, 42). To test if EspM binds the genomic region upstream of the *whiB6_MT_* gene, we expressed and purified 6×His-tagged EspM*_MT_* in E. coli (the *MT* subscript refers to the EspM protein from M. tuberculosis) (Fig. S2). We amplified the 500 bp upstream of the *whiB6* gene from M. tuberculosis Erdman and tested if EspM*_MT_* specifically bound the *whiB6_MT_* promoter region using EMSAs. Increasing concentrations of 6×His-EspM*_MT_* protein led to a specific mobility shift of the *whiB6_MT_* promoter probe and to a corresponding loss of free probe (Fig. 1D). Although bound *rpoA* probe was not observed at the highest concentrations of 6×His-EspM*_MT_* protein, the free probe was reduced, indicating weak binding at the highest protein concentrations. Together, these data indicate that EspM, from both M. marinum and M. tuberculosis, directly and specifically bound the *whiB6-espM* intergenic region.

*espM* is divergently transcribed from the *whiB6* gene and is immediately adjacent to the *esx-1* locus (Fig. 1A). EspM is a predicted conserved regulatory protein (42), but the corresponding function has not been investigated. The EspM*_MM_* protein is predicted to have an N-terminal forkhead-associated (FHA) domain (amino acids [aa] 32 to 89) and a C-terminal helix-turn-helix domain (Fig. 1E). We hypothesized that the C-terminal half of the protein mediated DNA binding. We expressed and purified 6×His-tagged EspM_NT_ (aa 1 to 133) and EspM_CT_ (aa 127 to 363) M. marinum proteins from E. coli (Fig. S2). We tested the ability of each protein to bind the *whiB6* promoter probe using EMSA. The 6×His-EspM_NT_ protein did not shift the mobility of the *whiB6* promoter probe (Fig. 1F). Incubation with increasing concentrations of the 6×His-EspM_CT_ protein caused a shift in mobility of the *whiB6* promoter probe and a loss of free probe. We conclude that the C-terminal half of the EspM protein is required for DNA binding.

### EspM is a conserved regulator of *whiB6* and *esx-1* gene expression.

**FIG 2:**
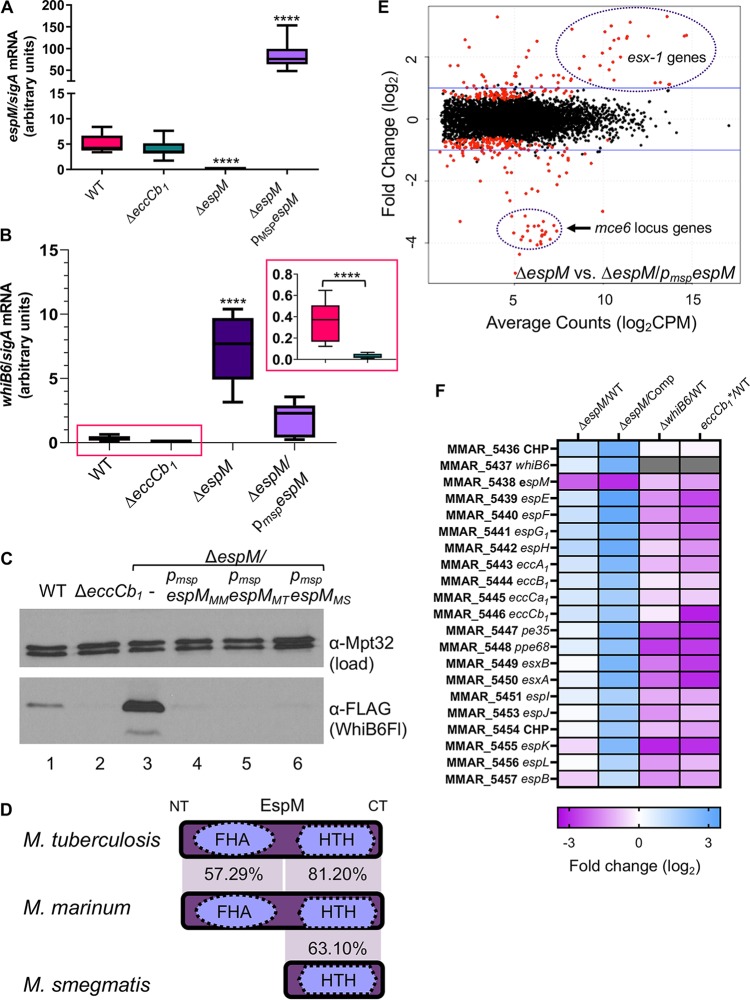
EspM is a conserved regulator of *whiB6* and *esx-1* gene expression. (A) qRT-PCR measuring the levels of *espM* expression relative to *sigA* expression. A one-way ordinary analysis of variance (ANOVA) (*P* < 0.0001), followed by a Dunnett’s multiple-comparison test relative to the WT strain, was performed. ****, *P* < 0.0001. (B) qRT-PCR measuring the levels of *whiB6* expression relative to *sigA* expression. A one-way ordinary ANOVA (*P* < 0.0001), followed by a Sidak’s multiple-comparison test relative to the WT strain, was performed. ****, *P* < 0.0001. The inset shows just the comparison between the WT and Δ*eccCb1* strains. A Student’s unpaired, two-tailed *t* test was used to define the significance of the results of the comparisons between the two strains. For panels A and B, the data represent averages of results from at least three biological replicates, each performed in technical triplicate. (C) Western blot analysis of 10 μg of protein per lane. Anti-Mpt32 was used as the loading control. All M. marinum strains indicated in this panel contained a C-terminal FLAG epitope tag on the *whiB6* gene. Samples were resolved on an 18% Tris-glycine gel. The Western blot shown is representative of at least three independent biological replicates. All strains indicated in panel D contained a C-terminal epitope tag on the *whiB6* gene. (D) Conservation of the EspM proteins (percent identity at the amino acid level) from M. tuberculosis, M. marinum, and M. smegmatis. (E) Scatterplot of genes differentially expressed in the Δ*espM* strain versus the Δ*espM*/*p_msp_espM* complemented strain. Genes indicated in red had a *q* value of <0.05. Regions enriched with the *esx-1* locus or *mce6* locus are highlighted. Full gene lists are available in Table S3. (F) Heat map of *esx-1* locus genes that are significantly differentially regulated in the Δ*espM* strain versus the Δ*espM*/*p_msp_espM* complemented strain compared to genes expressed in the Δ*espM*, Δ*whiB6*, or *eccCb_1_* mutant strains relative to the WT strain.

We confirmed that the *espM* transcript was absent in the Δ*espM* M. marinum strain using quantitative reverse transcription-PCR (qRT-PCR) (Fig. 2A). The *espM* expression level was significantly higher in the Δ*espM*/*p_msp_espM* complemented strain than in the wild-type (WT) strain (*P* < 0.0001). These data indicate that the complementation strain is an *espM* overexpression strain. We did not observe a significant reduction of *espM* gene expression in the Δ*eccCb_1_* strain relative to the WT strain. These data confirm that *espM* expression is not regulated by the ESX-1 system in M. marinum, consistent with our previously published transcriptomic analysis (35).

Because EspM bound the region upstream of the *whiB6* gene, we tested if EspM regulates *whiB6* gene expression. We measured *whiB6* gene expression in M. marinum using qRT-PCR. Consistent with our prior findings (35), *whiB6* gene expression was significantly reduced in the Δ*eccCb_1_* strain compared to the WT strain (Fig. 2B, inset, *P* < 0.0001). Deletion of the *espM* gene resulted in a significant increase in *whiB6* expression relative to the WT strain (*P* < 0.0001). Overexpression of the *espM* gene resulted in *espM* expression that was not significantly different from that seen with the WT strain. We conclude that EspM is a repressor of *whiB6* gene expression.

We measured the levels of WhiB6 protein in the presence and absence of the *espM* gene (Fig. 2C). The parental M. marinum strain for these strains includes a *whiB6* gene with a C-terminal FLAG epitope tag (WhiB6Fl [35]). Consistent with our previously published data (35), the WhiB6Fl protein was absent from the lysate generated from the Δ*eccCb_1_* strain (Fig. 2C, lane 2). Consistent with the expression data (Fig. 2B), deletion of the *espM* gene resulted in increased WhiB6Fl protein levels relative to those seen with the WT strain (Fig. 2C, compare lane 3 to lane 1). The WhiB6Fl protein levels in the Δ*espM*/*p_msp_espM_MM_* complemented strain (*espM* overexpression) were lower than those in the WT strain (Fig. 2C, lane 4 versus lane 1). Together, these data strongly support the conclusion that EspM represses *whiB6* gene expression in M. marinum.

The *espM* gene is syntenic in M. marinum, M. tuberculosis, *and* M. smegmatis (Fig. S3). M. smegmatis is a nonpathogenic, soil-dwelling mycobacterial species that uses the ESX-1 system to mediate conjugation (43, 44). The EspM orthologs in all three species are conserved at the protein level (Fig. 2D; see also Fig. S3). The M. smegmatis ortholog (MSMEG_0052; EspM*_MS_*) lacks the N-terminal FHA domain. Aligning the C-terminal halves of the EspM*_MM_* and EspM*_MT_* proteins with EspM*_MS_* revealed that the M. marinum and M. tuberculosis C-terminal halves are 81.20% identical at the amino acid level. EspM*_MS_* is 63.10% and 62.20% identical to the C-terminal half of EspM*_MM_* and EspM*_MT_*, respectively.

10.1128/mBio.02807-19.4 FIG S3 Conservation of the *espM* gene and protein in M. marinum, M. tuberculosis, and M. smegmatis. (A) *Rv3863* and *MSMEG_0052* are the *espM* orthologs in M. tuberculosis and M. smegmatis. The *esx-1* locus begins with the *espE* gene in M. marinum and M. tuberculosis and with the *espG* gene in M. smegmatis. (B) The alignment was generated using Clustal Omega, followed by visualization with BoxShade. Black, identity; gray, similarity. The asterisk (*) indicates conservation across all three species. The predicted FHA domain in M. marinum and M. tuberculosis is indicated in pink. The sequence of EspM_NT_ from M. marinum used as described for Fig. 1 ends at the red circle. EspM_CT_ from M. marinum used as described for Fig. 1 starts at the green arrow. Download FIG S3, PDF file, 2.6 MB. Copyright © 2020 Sanchez et al. 2020 Sanchez et al. This content is distributed under the terms of the Creative Commons Attribution 4.0 International license.

Because EspM proteins are conserved across three mycobacterial species, we hypothesized that the repression of *whiB6* expression by EspM would be functionally conserved. We generated integrating plasmids constitutively expressing the *espM* genes from M. tuberculosis Erdman (*espM_MT_*) and M. smegmatis mc^2^155 (*espM_MS_*) and introduced each plasmid into the Δ*espM* M. marinum strain. As shown in Fig. 2C, overexpression of the EspM*_MT_* protein or EspM*_MS_* protein reduced WhiB6Fl protein levels in the Δ*espM* M. marinum strain (Fig. 2C; compare lanes 5 and 6 with lane 3), similarly to the complemented strain overexpressing the *espM_MM_* gene (Fig. 2C, lane 4). These data demonstrate that repression of *whiB6* expression is functionally conserved between the EspM orthologs in M. marinum, M. tuberculosis, and M. smegmatis.

We performed RNA-seq transcriptional profiling to determine if EspM regulates other genes in addition to *whiB6* in M. marinum. Comparison of the WT strain to the *ΔespM* strain (both bearing the *whiB6Fl* allele) identified 134 genes that were upregulated and 300 genes that were downregulated (>2-fold; false-discovery rate [*q* value] of <0.05) (Fig. S4A; see also Table S3A). Genes controlled by EspM are also expected to be differentially regulated in the Δ*espM* strain compared to the complemented strain that overexpresses the repressor. We observed 44 genes that were upregulated and 55 genes that were downregulated in the Δ*espM* strain compared to the complemented strain (>2-fold; *q* value of <0.05) (Fig. 2E; see also Table S3B). Consistent with repression of *whiB6* expression by EspM, we observed that *whiB6* expression was induced 1.6-fold and 7.0-fold in the Δ*espM* strain compared to the WT strain and the complemented overexpression strain, respectively. Of the 44 genes that were induced in the Δ*espM* strain compared to the complemented strain, 21 genes from the *esx-1* locus were identified (*MMAR_5436* to *MMAR_5457*), including 8 genes that were also induced in the Δ*espM* strain compared to WT strain (Fig. 2F; see also Fig. S4B). Most of the other genes in the *esx-1* locus were significantly induced in the Δ*espM* strain relative to the WT strain, but with induction levels below 2-fold.

10.1128/mBio.02807-19.5 FIG S4 EspM broadly regulates gene expression in M. marinum and fine-tunes ESX-1 protein levels. (A) Scatter plot of genes differentially expressed in the Δ*espM* strain versus the WT strain. Genes highlighted in red had a *q* value of <0.05. (B) Venn diagram of genes that were induced (>2×, *q* value of <0.05) in the Δ*espM*/WT or Δ*espM*/complemented strain from this study or that were repressed (>2×, *q* value of <0.05) in the Δ*whiB6* or *eccCb1* ochre mutant (relative to the WT strain) from Bosserman et al. (35). (C) Heat map of genes in the *mce6* locus and of surrounding genes that were significantly downregulated (>2×, *q* value of <0.05) in the Δ*espM*/WT or Δ*espM*/complemented comparison. These genes were induced in the Δ*whiB6* or *eccCb1* ochre mutants relative to the WT strain, consistent with expression being *whiB6* and ESX-1 dependent; however, the induction in gene expression did not achieve statistical significance for these strains. All strains in this figure contained the *whiB6Fl* allele at the *whiB6* locus. (D) Western blot analysis of EsxA production in M. marinum lysates. The strains are the same as those shown in Fig. 5. A 10-μg or 5-μg volume of protein, as indicated, was loaded per lane and resolved on a 4% to 20% gel. Mpt-32 served as a loading control. The image shown is representative of results of three biological replicates. (E) Relative quantification of ESX-1-associated proteins using label-free proteomics. The proteins were derived from the strains described in the Fig. 5 legend. All protein levels are represented as log2-fold changes compared with those measured in the WT (*whiB6Fl*) strain or the Δ*espM* strain. Only the subset of ESX-1-associated proteins that were reproducibly quantified are shown in this heat map. The complete data are presented in Table S1E and F. The data shown are representative of results from one biological replicate, performed in technical duplicate. (F) Thin-layer chromatography of lipids extracted from the strains described in the Fig. 5 legend. PDIM purified from M. tuberculosis H37Rv was used as a positive control. TAG is triacylglycerols. The *R_f_* values are as follows: for M. tuberculosis PDIM and the WT, *espM*, and *eccCb*_1_ strains, 3.17 cm; for the complemented strain, 3.07 cm, likely due to the slight curvature of the band. The results shown are representative of experiments performed in biological triplicate. Download FIG S4, PDF file, 0.8 MB. Copyright © 2020 Sanchez et al. 2020 Sanchez et al. This content is distributed under the terms of the Creative Commons Attribution 4.0 International license.

We also observed induction of unlinked *esx*-associated loci in the Δ*espM* strain compared to the complemented strain, including *MMAR_0187-188* (*esxB*_*1esxA_1*), MMAR_3654 (*esxP2*), and the ESX-1 substrate locus *MMAR_2894* (45) (Table S3B). Several of these genes were previously shown to be regulated by WhiB6 or ESX-1 (Fig. 2F; see also Fig. S4B).

Of the 55 genes downregulated in the Δ*espM* strain relative to the complemented strain, 39 were also downregulated in the Δ*espM* strain relative to WT strain (Table S3). These included 24 strongly downregulated genes between *MMAR_0159* and *MMAR_0182* (Fig. 2E; see also Table S3B), which includes the *mce6* locus (Fig. S4C), and genes for amino acid metabolism and lipid anabolism. Prior studies with the Δ*whiB6* and *eccCb_1_* mutant strains showed induction of the genes in the *mce6* locus (Fig. S4C), supporting the idea of ESX-1-dependent regulation. Curiously, we also detected downregulation of an ESX-1-associated operon, *MMAR_4166* to *MMAR_4168* (*espA*, *espC*, and *espD*). Together, these data strongly support the conclusion that EspM is a regulator of genes broadly associated with the ESX-1 system in M. marinum.

### EspM represses *whiB6* expression in the absence of the ESX-1 membrane complex.

**FIG 3:**
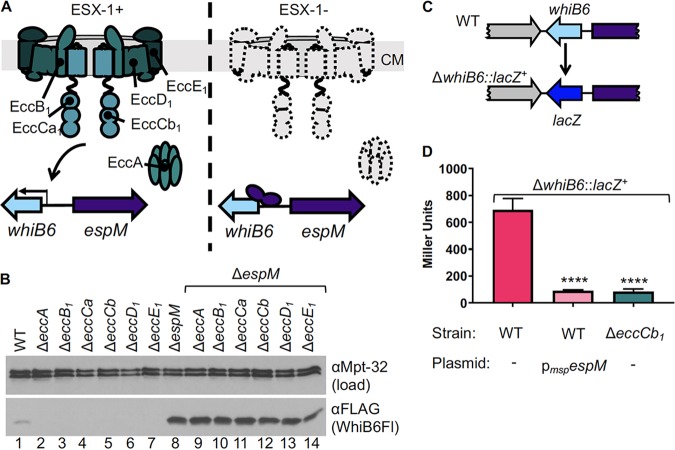
EspM is required for the repression of *whiB6* expression in the absence of the ESX-1 membrane complex. (A) Schematic of the ESX-1 membrane complex and the proposed role of EspM in ESX-1-dependent gene expression. CM, cytoplasmic membrane. The depiction of the membrane complex was adapted from reference 28. (B) Western blot analysis of 10 μg per lane on an 18% gel. Mpt-32 was used as a loading control. The image is representative of three independent biological replicates. All strains indicated in panels B and C contained a C-terminal epitope tag on the *whiB6* gene. (C) Schematic of the Δ*whiB6*::*lacZ^+^* reporter strain. (D) β-Galactosidase assay in WT M. marinum strains. The data in the figure represent averages of results from four independent biological replicates, each performed in technical triplicate. Significance was determined using a one-way ordinary ANOVA (*P* < 0.0001) followed by a Tukey’s multiple-comparison test. ****, *P* < 0.0001. The WT/p*_msp_espM* and Δ*eccCb_1_* levels were not significantly different from each other (*P* = 0.9694). Error bars represent standard errors.

*whiB6* gene expression levels are reduced in the absence of the ESX-1 membrane complex (35). We hypothesized that EspM represses *whiB6* and *esx-1* gene expression in the absence of the ESX-1 membrane complex (Fig. 3A). Four ESX-conserved components (Ecc’s) reside in the CM (Fig. 3A; EccB_1_, EccCa_1_, EccD_1_ and EccE_1_ [28, 29]), and two Ecc’s (EccCa_1_ and EccA) are cytoplasmic (10, 46–49).

We reasoned that if EspM repressed *whiB6* gene expression in the absence of the ESX-1 membrane complex, then deletion of the *espM* gene in strains lacking the ESX-1 membrane complex would restore *whiB6* gene expression. We generated M. marinum strains bearing deletions of each *ecc* gene (*eccA* to *eccE_1_*) alone or in combination with deletion of the *espM* gene. Deletion of any *ecc* gene resulted in a loss of WhiB6Fl protein relative to the WT strain (Fig. 3B, lanes 2 to 7 versus lane 1). The deletion of the *espM* gene in combination with the *ecc* genes (Δ*espM* Δ*eccA*, Δ*espM* Δ*eccB_1_*, Δ*espM* Δ*eccCa_1_*, Δ*espM* Δ*eccCb_1_*, Δ*espM* Δ*eccD_1_*, and Δ*espM* Δ*eccE_1_* mutant strains) resulted in levels of WhiB6Fl similar to those in the Δ*espM* strain (Fig. 3B, lanes 9 to 14 versus lane 8) and higher than those in the WT strain (Fig. 3B, lane 1). We further demonstrated that complementation with the *eccA* gene or the *espM* gene in the Δ*espM* Δ*eccA* strain resulted in levels of WhiB6Fl similar to those seen with the Δ*espM* deletion strain or the Δ*eccA* deletion strain, respectively (Fig. S5).

10.1128/mBio.02807-19.6 FIG S5 EspM is required for *whiB6* repression in the Δ*eccA* M. marinum strain. Western blot analysis of 10 μg per lane on an 18% gel was performed. Mpt-32 was used as a loading control. The image is representative of results from three independent biological replicates. All strains contained a C-terminal epitope tag on the *whiB6* gene. Download FIG S5, PDF file, 0.2 MB. Copyright © 2020 Sanchez et al. 2020 Sanchez et al. This content is distributed under the terms of the Creative Commons Attribution 4.0 International license.

We reasoned that overexpression of the *espM* gene might be sufficient to repress *whiB6* expression to levels similar to those seen with the Δ*eccCb_1_* strain. We generated a strain bearing a *whiB6* transcriptional reporter in M. marinum. We replaced the *whiB6* gene with the *lacZ* gene, creating a strain lacking the *whiB6* gene and with a reporter fusion to the *whiB6* promoter (Δ*whiB6*::*lacZ^+^*; Fig. 3C). We generated an isogenic Δ*eccCb_1_* strain (no ESX-1 membrane complex [30]), as well as an isogenic WT strain overexpressing the *espM_MM_* gene. The level of β-galactosidase activity was significantly reduced in the Δ*eccCb_1_* strain compared to the WT strain (Fig. 3D; *P* < 0.0001), confirming that the *whiB6*::*lacZ^+^* reporter fusion was regulated by the ESX-1 membrane complex (35). Overexpression of the *espM* gene in the WT strain significantly reduced the levels of β-galactosidase activity compared to the WT strain levels (*P* < 0.0001). The levels of β-galactosidase activity in the Δ*eccCb_1_* and *espM* overexpression strains were not significantly different from each other (*P =* 0.9915), demonstrating that overexpression of *espM* is sufficient to repress *whiB6* gene expression in M. marinum. Collectively, our data demonstrate that EspM is required for repression of *whiB6* gene expression in the absence of the ESX-1 membrane complex. Moreover, because the reporter strain lacks the *whiB6* gene, these data indicate that EspM represses *whiB6* expression in a WhiB6-independent manner.

### The EspM and WhiB6 regulators coordinately control gene expression.

**FIG 4:**
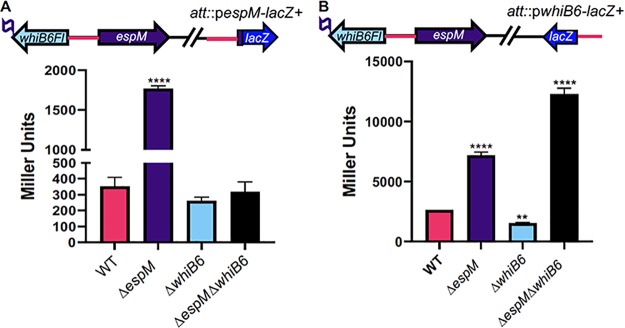
EspM and WhiB6 mutually regulate the expression of the *espM* and *whiB6* genes. (A) The *whiB6*/*espM* locus and the *espM-lacZ^+^* transcriptional fusion integrated at the *attB* site in M. marinum. The flag at the C terminus of the *whiB6* gene indicates the presence of the *whiB6Fl* allele. Data represent β-galactosidase activity of the *espM-lacZ^+^* transcriptional fusion. Error bars represent propagated errors. A one-way ordinary ANOVA (*P* < 0.0001) followed by a Sidak’s multiple-comparison test was performed. Significance is shown relative to the WT strain. ****, *P* < 0.0001. (B) The *whiB6*/*espM* locus and the *whiB6-lacZ^+^* transcriptional fusion integrated at the *attB* site in M. marinum. Data represent β-galactosidase activity of the *whiB6-lacZ^+^* transcriptional fusion. Error bars represent propagated errors. A one-way ordinary ANOVA (*P* < 0.0001) followed by a Tukey’s multiple-comparison test was performed. Significance data shown are relative to the WT strain. ****, *P* < 0.0001; **, *P* = 0.0078. For both panels, the data represent averages of results from at least three biological replicates, each performed in technical triplicate. All strains indicated in Fig. 4, with the exception of the Δ*whiB6* and Δ*whiB6* Δ*espM* strains, contained a C-terminal epitope tag on the *whiB6* gene.

The *espM* and *whiB6* genes are divergently organized in mycobacterial genomes (Fig. 4A). Because the *whiB6* and *espM* genes share an intergenic region which likely controls the expression of both genes (Fig. 4A, pink), we sought to further define the relationship between the EspM and WhiB6 regulators.

We generated *espM-lacZ^+^* and *whiB6-lacZ^+^* integrating transcriptional reporters (Fig. 4). The *espM-lacZ^+^* reporter resulted in significantly increased β-galactosidase activity in the Δ*espM* strain compared to the WT strain (*P* < 0.0001; Fig. 4A). Loss of the *whiB6* gene did not significantly impact β-galactosidase activity relative to the WT strain (*P* = 0.1195). Deletion of both the *espM* and *whiB6* genes (Δ*espM* Δ*whiB6* mutant strain) resulted in β-galactosidase activity comparable to that seen with the WT M. marinum strain (*P* = 0.9305). We conclude from these data that *espM* expression is negatively autoregulated. Moreover, in the absence of EspM, WhiB6 is required for the observed increased *espM* gene expression. We confirmed that WhiB6 binds the *whiB6-espM* intergenic region by expressing and purifying a C-terminally 6×His-tagged WhiB6*_MM_* fusion protein from Escherichia coli (Fig. S2) and performing EMSAs (Fig. S6). We observed a specific shift in mobility of the *whiB6* promoter probe (Fig. 1A, “EMSA probe”) and a concomitant loss of free *whiB6* promoter probe with increasing concentrations of the WhiB6*_MM_*-6×His protein (Fig. S6). We did not observe a mobility shift of the *rpoA* probe, confirming the specific binding of the WhiB6*_MM_* protein to the *whiB6* promoter probe.

10.1128/mBio.02807-19.7 FIG S6 WhiB6 binds the *whiB6-espM* intergenic region *in vitro.* EMSA was performed with increasing concentrations of WhiB6 protein from M. marinum. The *whiB6* promoter probe was the same as that used as described in the Fig. 1 legend (bp 6577396 to 6577960 in the M. marinum M genome; 30 bp upstream of the *whiB6* ORF to 141 bp into *MMAR_5438*). The control probe was 500 bp of the *rpoA* ORF (bp 1309999 to 1310499). Download FIG S6, PDF file, 0.7 MB. Copyright © 2020 Sanchez et al. 2020 Sanchez et al. This content is distributed under the terms of the Creative Commons Attribution 4.0 International license.

The presence of the *whiB6-lacZ^+^* reporter resulted in significantly increased β-galactosidase activity in the Δ*espM* strain compared to the WT strain (*P* < 0.0001; Fig. 4B). Loss of the *whiB6* gene caused a significant reduction in β-galactosidase activity relative to the WT strain (*P* = 0.0078). Deletion of the *espM* and *whiB6* genes together (Δ*espM* Δ*whiB6* mutant strain) resulted in significantly increased β-galactosidase activity relative to the WT and Δ*espM* M. marinum strains (*P* < 0.0001 for both comparisons). This further supports the idea that EspM represses *whiB6* gene expression and confirms positive autoregulation of WhiB6, consistent with prior findings (38). Moreover, the significant increase in *whiB6* expression in the absence of both EspM and WhiB6 suggests there is at least one more transcriptional activator of *whiB6* expression in M. marinum.

### EspM fine-tunes ESX-1 function in M. marinum.

**FIG 5:**
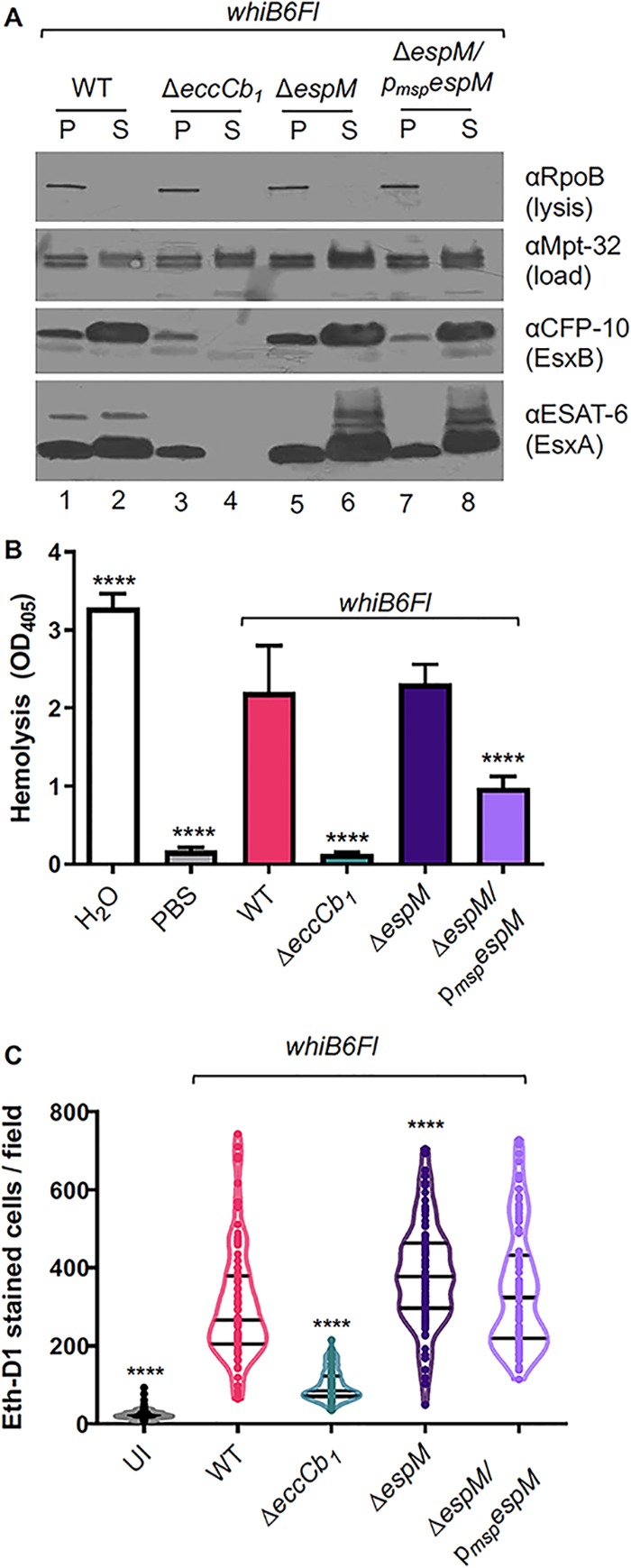
EspM fine-tunes ESX-1 function. (A) Western blot analysis of EsxA and EsxB secretion *in vitro*. 10 μg of protein was loaded per lane and resolved on a 4% to 20% gel. RpoB was used as the lysis control; Mpt-32 is a Sec-dependent secreted protein that served as a loading control. The image shown is representative of three biological replicates. (B) Hemolysis assay of M. marinum strains. The image shown represents at least three biological replicates, each performed in technical triplicate. Error bars represent the propagated errors. A one-way ordinary ANOVA (*P* < 0.0001) followed by a Tukey’s multiple-comparison test was performed. ****, *P* < 0.0001 (relative to the WT strain). OD_405_, optical density at 405 nm. (C) Cytolysis assay of RAW 264.7 cells following 24 h of infection with M. marinum at an MOI of 7. Black bars indicate median and quartiles. UI, uninfected. Statistical analysis was performed using a one-way ordinary ANOVA (*P* < 0.0001) followed by a Tukey’s multiple-comparison test. ****, *P* < 0.0001 (compared to the WT strain). Each dot represents the number of EthD-1-stained cells in a single field. A total of 10 fields were counted using ImageJ for each well. Processing of 3 wells was performed for each biological replicate. A total of 90 fields were counted for each strain.

Because EspM regulates *whiB6* and *esx-1* gene expression, we tested if EspM was required for ESX-1 activity. The WT strain produced the EsxA and EsxB substrates and secreted them into the culture supernatant during growth *in vitro* (Fig. 5A, lanes 1 and 2). Deletion of the *eccCb_1_* gene, which is required for ESX-1 secretion (10, 12, 14), reduced production of EsxA and EsxB, and neither protein was secreted (Fig. 5A, lanes 3 and 4). The Δ*espM* strain exhibited at least WT levels of production and secretion of EsxA and EsxB (Fig. 5A, lanes 5 and 6). The *espM* complemented strains showed reduced levels of production of EsxA and EsxB (Fig. 5A, lanes 7 and 8, and Fig. S4D) but exhibited at least wild-type levels of secretion of EsxA and EsxB. To further confirm that the levels of ESX-1 proteins were altered, consistent with the observed EspM-dependent expression changes, we performed global proteomics on whole-cell lysates of the M. marinum strains represented in panel A (for the WT, Δ*eccCb_1_*, Δ*espM*, and complemented strains) (Table S1E and F; see also Fig. S4E). We identified 1,881 proteins at a 1% false-discovery rate. Protein quantification was performed by using label-free quantification (LFQ). We found that, similarly to the EsxA and EsxB proteins, the levels of several ESX-1 substrates (EspF, EspK, and EspB) and components (EccA) and other associated proteins (EspG, EspH, and EspL) were significantly reduced in the complemented strain, consistent with the expression data (Fig. 2). These data demonstrate that EspM is required for fine-tuning the levels of ESX-1-associated proteins, including the EsxA and EsxB substrates, in the mycobacterial cells but not for the secretory function of the ESX-1 system.

The ESX-1 system promotes phagosomal lysis during macrophage infection (6, 9, 27). M. marinum lyses red blood cells (RBCs) in an ESX-1-dependent manner (14, 17, 50). Hemolysis analysis is a common way to measure the membranolytic activity of the ESX-1 system (14, 17, 50). The WT strain caused significantly increased hemolytic activity compared to the phosphate-buffered saline (PBS) control (no bacteria) (*P* < 0.0001; Fig. 5B). The Δ*eccCb_1_* strain exhibited hemolytic activity that was not significantly different from that seen with the PBS control (*P =* 0.9996). The Δ*espM* strain exhibited hemolytic activity not significantly different from that seen with the WT strain (*P =* 0.9602). The complemented strain, which overexpresses *espM* relative to the WT strain (Fig. 2B), showed significantly reduced hemolytic activity relative to the WT and Δ*espM* strains (*P* < 0.0001).

ESX-1-deficient M. marinum strains fail to lyse the phagosome and fail to lyse macrophages (7, 51). We infected RAW 264.7 cells with M. marinum at a multiplicity of infection (MOI) of 7 and measured macrophage lysis by visualizing and quantifying the uptake of ethidium homodimer by permeabilized macrophages (52). Consistent with previous findings (51, 53), the WT strain caused macrophage lysis (Fig. 5C). Infection with the Δ*eccCb_1_* strain resulted in a significant reduction in macrophage lysis compared to infection with the WT strain (*P* < 0.0001). In contrast, infection with the Δ*espM* strain resulted in significantly increased macrophage lysis compared to infection with the WT strain (*P* < 0.0001). Infection with the *espM* overexpression strain restored macrophage lysis to WT levels (*P* = 0.2138). These data show that in the absence of the *espM* gene, the ESX-1 system promoted higher levels of macrophage lysis. Moreover, combined with the hemolysis data, these findings indicate that the levels of EspM fine-tune the activity of the ESX-1 system in M. marinum.

## DISCUSSION

Collectively, our findings identify EspM as a conserved transcription factor required for the ESX-1-dependent transcriptional response in pathogenic mycobacteria. Although the *espM* gene is adjacent to the *esx-1* locus, EspM has not been previously characterized. The *espM* gene may not have been linked to the ESX-1 system previously because deletion of the *espM* gene in M. marinum had only a subtle impact on ESX-1 activity, likely because several transcription factors regulate *whiB6* gene expression (39, 54–56). Moreover, the M. tuberculosis EspM ortholog Rv3863 was previously reported to be essential for growth *in vitro* in some genome-wide studies (57, 58) and nonessential in others (59), which may complicate study in M. tuberculosis.

**FIG 6:**
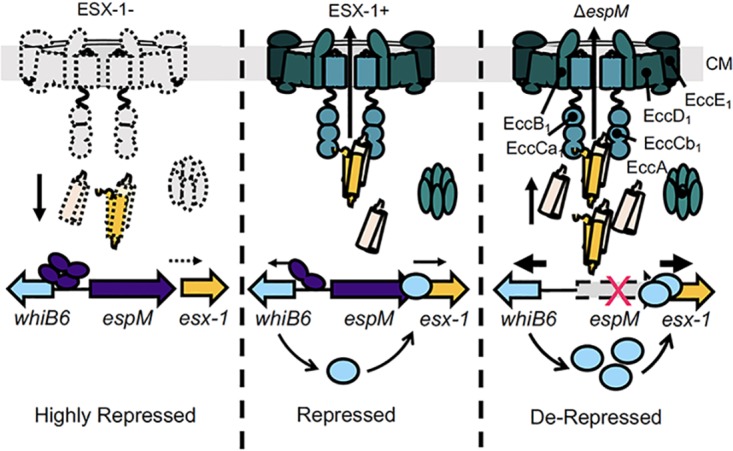
EspM regulates gene expression in response to the ESX-1 system. A model proposing a role for EspM in regulating *whiB6* gene expression is shown. In the absence of the ESX-1 membrane complex, we propose that EspM represses *whiB6* gene expression (highly repressed state). Reduced WhiB6 levels cause reduced activation of ESX-1 substrate gene expression, preventing substrate accumulation. In the presence of ESX-1, EspM still promotes repression of *whiB6* gene expression, fine-tuning expression of the *esx-1* substrate genes (repressed state). In the absence of *espM*, the levels of *whiB6* and *esx-1* substrate gene expression are derepressed.

The identification of EspM further expands our understanding of the feedback control mechanism that links the levels of ESX-1 substrates, and other genes, to the assembly of the secretory apparatus (35). We found that deletion of the *espM* gene resulted in levels of *whiB6* expression that were higher than those seen with the WT strain (Fig. 2B and C). We propose that *whiB6* expression is repressed by EspM in the WT strain and that *whiB6* gene expression is further repressed by EspM in the absence of the ESX-1 system (Fig. 6). Therefore, regulation by EspM is relevant in WT bacteria and not simply when the ESX-1 system is absent, which may or may not be physiologically relevant.

We do not yet understand how the ESX-1 membrane complex controls the magnitude of *whiB6* repression by EspM. We do not think that the ESX-1 system transcriptionally regulates the *espM* gene. We observed no ESX-1-dependent change in *espM* transcript levels either here (Fig. 2A) or in our prior work (35). These findings contrast those of Abdallah et al. (36), which indicated that the *Rv3863* transcript (*espM_MT_*) is regulated by the ESX-1 system in M. tuberculosis, similarly to the *whiB6* gene. This may be an example of differential regulation between M. marinum and M. tuberculosis. The presence or absence of an assembled ESX-1 membrane complex likely posttranscriptionally controls the levels of EspM in M. marinum. We recapitulated the levels of *whiB6* gene expression in the Δ*eccCb_1_* strain by overexpressing *espM* in the WT strain (Fig. 3D). However, our published proteomic data indicate that EspM*_MM_* protein levels are reduced 2-fold in the absence of EccCb_1_, when repression of *whiB6* expression is strongest (35). Regulation of the EspM transcription factor may be similar to the control of gene expression by type III secretion systems (T3SS) in Gram-negative bacteria (60–63). The injectisome T3SS uses cytoplasmic substrates and/or chaperones to posttranscriptionally modulate the levels or activity of transcription factors that regulate secretion-associated genes (62, 64–69). ESX-1 substrates or chaperones may posttranscriptionally regulate the activity of EspM in response to the presence or absence of the ESX-1 membrane complex.

Posttranscriptional regulation of EspM could occur through the predicted N-terminal forkhead-associated (FHA) domain. FHA domain-containing proteins posttranscriptionally regulate Gram-negative type III and type VI protein secretion systems (70–72). Staphylococcus aureus has an Ess-type VII secretion system similar to the ESX-1 system (73, 74). The EccC-related protein EssC*_SA_* (75, 76) includes a twin-FHA domain that is essential for secretion (77). FHA domains also mediate oligomerization (78–80). We observed a second shift in mobility of the *whiB6-espM* probe by EMSA with increasing concentrations of EspM*_MM_* protein (Fig. 1C and F). We did not observe this supershifted product when using the C-terminal half of EspM*_MM_* alone (Fig. 1F), suggesting that the N-terminal half of the protein is important for this observation. The FHA domain may directly or indirectly control oligomerization of EspM in response to the ESX-1 membrane complex.

Although WhiB6 directly binds the *whiB6-espM* promoter region, we did not identify WhiB6 in the DNA pulldown (Fig. 1; see also Table S1 in the supplemental material). We have not routinely identified WhiB6 from M. marinum lysates using mass spectrometry. We also did not identify the PhoP response regulator, which regulates *whiB6* expression in M. tuberculosis. Under the conditions of our experiments, EspM may bind the intergenic region preferentially to other regulators, including WhiB6 and PhoP. This idea is supported by the finding that WhiB6 activates *espM* gene expression only in the absence of EspM (Fig. 4A). Also, it is possible that no single technique can identify all proteins that bind and regulate a specific region. For example, chromatin immunoprecipitation sequencing (ChIP-seq) experiments in strains overexpressing WhiB6 in M. tuberculosis did not identify direct binding of WhiB6 to the *whiB6-espM* intergenic region. And yet, overexpression of WhiB6 resulted in a significant upregulation of *whiB6* gene expression in the same study (55, 56). Likewise, although PhoP bound the WhiB6 promoter directly in M. tuberculosis, overexpression of PhoP failed to significantly impact *whiB6* gene expression (55, 56). Therefore, the absence of enrichment of regulators in our study does not preclude the possibility of a role for them in the regulation of *whiB6* and *espM* expression. Finally, regulation of the *whiB6* and *espM* genes may not be conserved between M. marinum and M. tuberculosis. In the case of *whiB6* expression, it has already been established that there is variability in how PhoP regulates *whiB6* in M. tuberculosis strains (39). It has not yet been established if *whiB6* regulates *esx-1* in M. marinum as part of the PhoPR regulon.

The back-to-back divergent arrangement of two regulators is a common theme in microorganisms (81), the best described of which are the cI and Cro regulators of bacteriophage λ (82). Divergence in organization allows tight coordination of the expression of both transcription factors and of their regulons from a single genetic locus. The intergenic region between the *espM* and *whiB6* genes likely contains binding sites for both WhiB6 and EspM. Indeed, we demonstrated using EMSAs that both EspM and WhiB6 bind this region *in vitro* (Fig. 1; see also Fig. S6 in the supplemental material) and that both contribute to regulating the *whiB6* and *espM* genes [Fig. 4]) (38, 39). Therefore, the genes regulated by WhiB6, the ESX-1 system, and EspM may be coordinated to fine-tune the ESX-1 secretion and for additional biological purposes. Moreover, whereas approximately half of the genes induced or repressed in the Δ*espM* strain versus the complemented strain are associated with the ESX-1 system, other EspM-regulated genes may have a currently unrecognized role in ESX-1-associated functions.

Our data clearly demonstrate that EspM impacts the expression of *esx-1-*associated genes and is associated with corresponding changes in ESX-1 protein levels, supporting the idea that EspM functions to fine-tune ESX-1 function. Consistent with these findings, we observed reduced hemolytic activity upon overexpression of *espM* and increased cytolytic activity in the absence of EspM. Although hemolysis and macrophage cytolysis are both measures of ESX-1 function, our prior work indicated that the results of the two assays do not always align, especially when using strains with intermediate ESX-1 production or secretion levels (45). It is possible that there are additional roles for EspM in *ex vivo* infection that differ from those seen in our studies *in vitro*. Alternatively, EspM could impact the expression of additional genes required for phagosomal lysis or macrophage cytolysis. For example, phthiocerol dimycocerosate (PDIM) has been implicated in both phagosomal lysis and macrophage cytolysis (83, 84). However, we did not see changes in the expression of genes required for PDIM synthesis and transport in our RNA sequencing analysis (Table S3) or in the production of PDIM (Fig. S4F).

Unlike most examples of T3SS-dependent gene expression, the genes regulated by EspM and the ESX-1 membrane complex are not restricted to the ESX-1-associated genes (35, 36). The C. trachomatis T3SS, which impacts global gene expression, may represent a temporal cue for regulating gene expression during infection (85). Likewise, the assembly of the ESX-1 system may serve as a temporal cue to regulate mycobacterial gene expression. While pathogenic mycobacteria elicit a transcriptional response essential for survival in the phagosome (1, 4), there has been no report of a transcriptional response to interaction with the macrophage cytosol. The cytoplasm is considered restrictive for bacterial survival and growth unless the pathogen adapts (86). Listeria monocytogenes, a pathogen that lyses the phagosomal membrane and accesses the cytoplasm (87), adapts by altering metabolism and inducing stress response pathways (86). We propose that the assembly of the ESX-1 membrane complex elicits gene expression pathways to link ESX-1-mediated phagosomal lysis and cytoplasmic adaptation. Indeed, several of the genes regulated by EspM and by the ESX-1 system are predicted to be associated with metabolism (Table S3). This is most notable in genes that are downregulated in the Δ*espM* strain or upregulated in the Δ*whiB6* and *eccCb_1_* mutant strains. For example, the genes in the *mce6* locus and surrounding genes were significantly downregulated in the Δ*espM* strain but were upregulated in the Δ*whiB6* and *eccCb_1_* mutant strains (Fig. S4C), although, due to variability in the data, the results representing the gene induction in the Δ*whiB6* and *eccCb_1_* mutant strains were not statistically significant. These data are supportive of the conclusion that the *mce6* genes are repressed in a *whiB6*-dependent manner, although further characterization studies will be required to support this hypothesis. *mce* genes have been associated with carbon nutrient uptake, including *mce1*, promoting uptake of fatty acids (88), and *mce4*, promoting uptake of cholesterol (89). *mce6* is absent in the M. tuberculosis genome but is present in the genomes of many nontuberculous mycobacterial species (90) and could play a role in controlling metabolite import to promote survival in the phagosome or cytosol. The *mce6* locus may be important for the cytosolic lifestyle of M. marinum, which polymerizes host actin and exhibits cytosolic motility (5), which is not conserved in M. tuberculosis.

Finally, because EspM regulates a subset of genes controlled by the ESX-1 system, there are likely additional transcription factors that make up an ESX-1-dependent transcriptional network. We focused on proteins that specifically bound the *whiB6*/*espM* intergenic region. Studies aimed at identifying proteins that bind additional ESX-1-responsive promoters would identify additional transcription factors in the ESX-1-responsive network.

In conclusion, we have identified a conserved transcription factor, EspM, which is encoded by a gene adjacent to the *esx-1* locus that is required for the repression of *whiB6* gene expression in the absence of the ESX-1 system. Our study results begin to define a transcriptional network that links the assembly of the ESX-1 system to widespread changes in gene expression, including the regulation of the ESX-1 apparatus and substrates.

## MATERIALS AND METHODS

A fully detailed explanation of the methods used in this study can be found in Text S1 in the supplemental material. All M. marinum strains were derived from M. marinum strain M (BAA-535). Where indicated, the parental strain included a FLAG epitope tag at the C terminus of the *whiB6* gene (35). Maintenance of the M. marinum strains and E. coli strains is described in Text S1. Enriched proteins were analyzed using quantitative nano-high-performance liquid chromatography–tandem mass spectrometry (nano-UHPLC-MS/MS) proteomics. All mycobacterial strains were generated using the allelic exchange protocol developed by Parish and Stoker (91) as described previously (35, 45, 52, 92). All strains, constructs, and primers (IDT, Coralville, IA) used in this study are listed in Table S2 in the supplemental material. All plasmids and genetic deletions were confirmed by targeted DNA sequencing performed at the Notre Dame Genomics and Bioinformatics Facility. All proteins were expressed in E. coli with 6×His affinity tags and purified using metal chelation affinity chromatography as described in Text S1. EMSAs were performed as reported previously (93–95), with modifications listed in Text S1. β-Galactosidase assays on M. marinum strains bearing the *whiB6*::*lacZ^+^*, *attB*::p*whiB6-lacZ^+^*, or *attB*::p*espM-lacZ^+^* reporter were performed as described previously (52). Hemolysis assays were performed as described previously (35). ESX-1 secretion assays were performed as described previously (35), except that 10 μg of protein was analyzed for all protein fractions. Western blot analysis was performed as described previously (35). Macrophage (RAW 264.7 cells) infections were performed as described previously (45) at an estimated multiplicity of infection (MOI) of 7 (2.5 × 10^6^ cells/ml). Cells were imaged and ethidium-homodimer uptake by perforated cells was quantified using ImageJ (35, 52). For transcriptional profiling, M. marinum strains were grown and RNA was extracted exactly as described previously (35). RNA sequencing was conducted as described previously (96), and the results were analyzed using SPARTA software (97). For analysis of differentially expressed genes (>2-fold; *q* value of <0.05), lists were filtered for genes with average counts greater than 4 (log_2_ CPM), with full unfiltered data sets available in Table S3.

10.1128/mBio.02807-19.9 TABLE S2 M. marinum strains, plasmids, and primers used in this study. Download Table S2, PDF file, 1.1 MB. Copyright © 2020 Sanchez et al. 2020 Sanchez et al. This content is distributed under the terms of the Creative Commons Attribution 4.0 International license.

10.1128/mBio.02807-19.10 TABLE S3 Gene expression tables presenting results of the RNA sequencing analysis performed in this study. (A) Gene expression tables for the Δ*espM* strain versus the WT (*whiB6Fl*) strain. (B) Gene expression tables for the Δ*espM* strain versus the complemented strain. Download Table S3, XLSX file, 2.0 MB. Copyright © 2020 Sanchez et al. 2020 Sanchez et al. This content is distributed under the terms of the Creative Commons Attribution 4.0 International license.

### Data availability.

The transcriptional profiling data are available at the NCBI GEO database (accession number GSE135072). All statistical analysis was performed as described in each figure legend, using PRISM v8.1.
