# Supplementary figures and images for: EspM Is a Conserved Transcription Factor That Regulates Gene Expression in Response to the ESX-1 System
Source: mBio. 2020 Feb 4;11(1):e02807-19. doi: 10.1128/mBio.02807-19 (PMC7002343; doi:10.1128/mBio.02807-19)

Figure S1

A

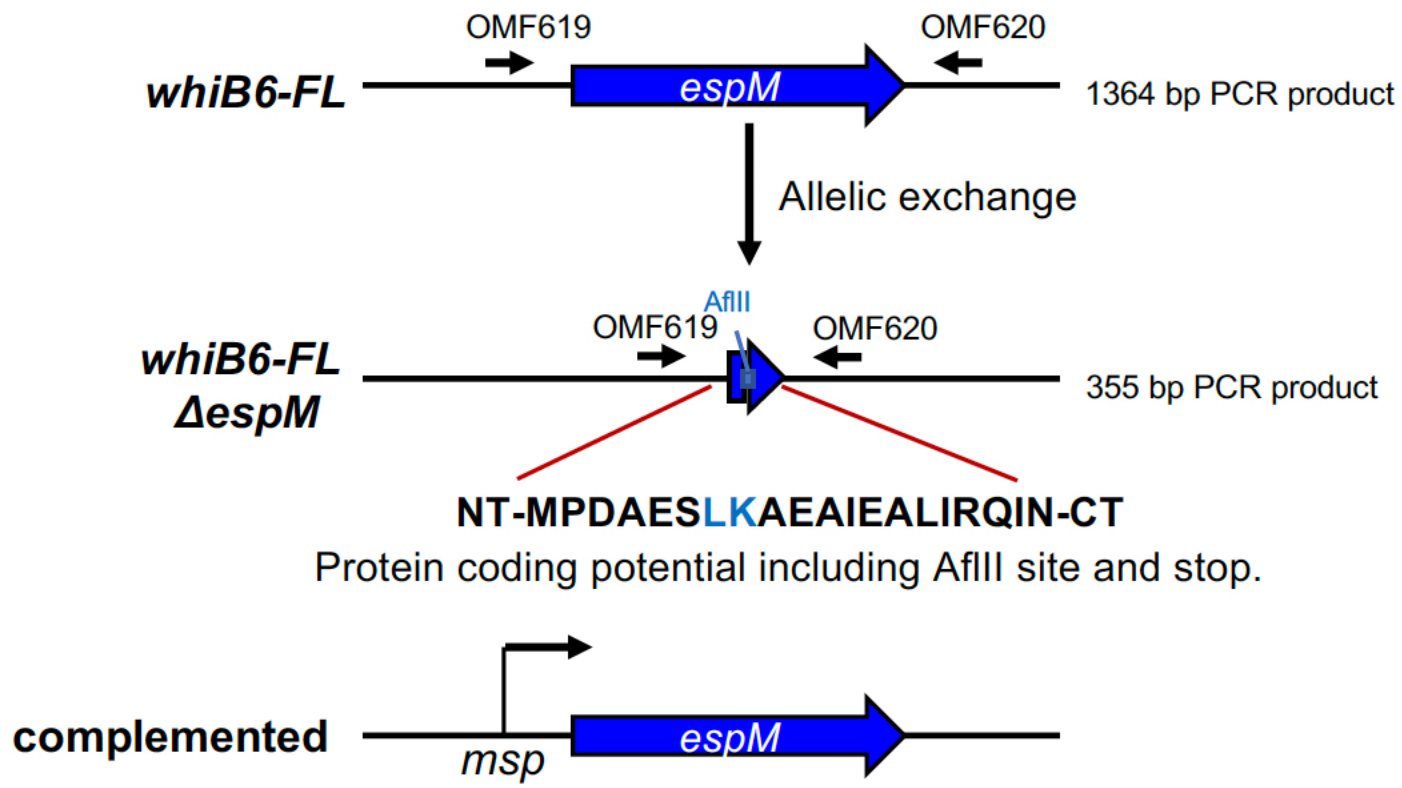

B

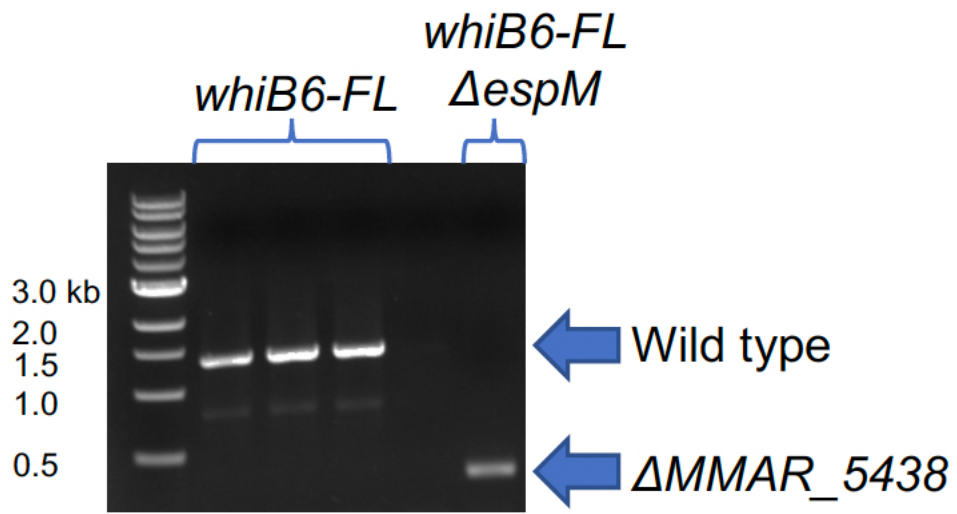

Supplement: FIG S1 [file mBio.02807-19-sf001.pdf]

Figure S2

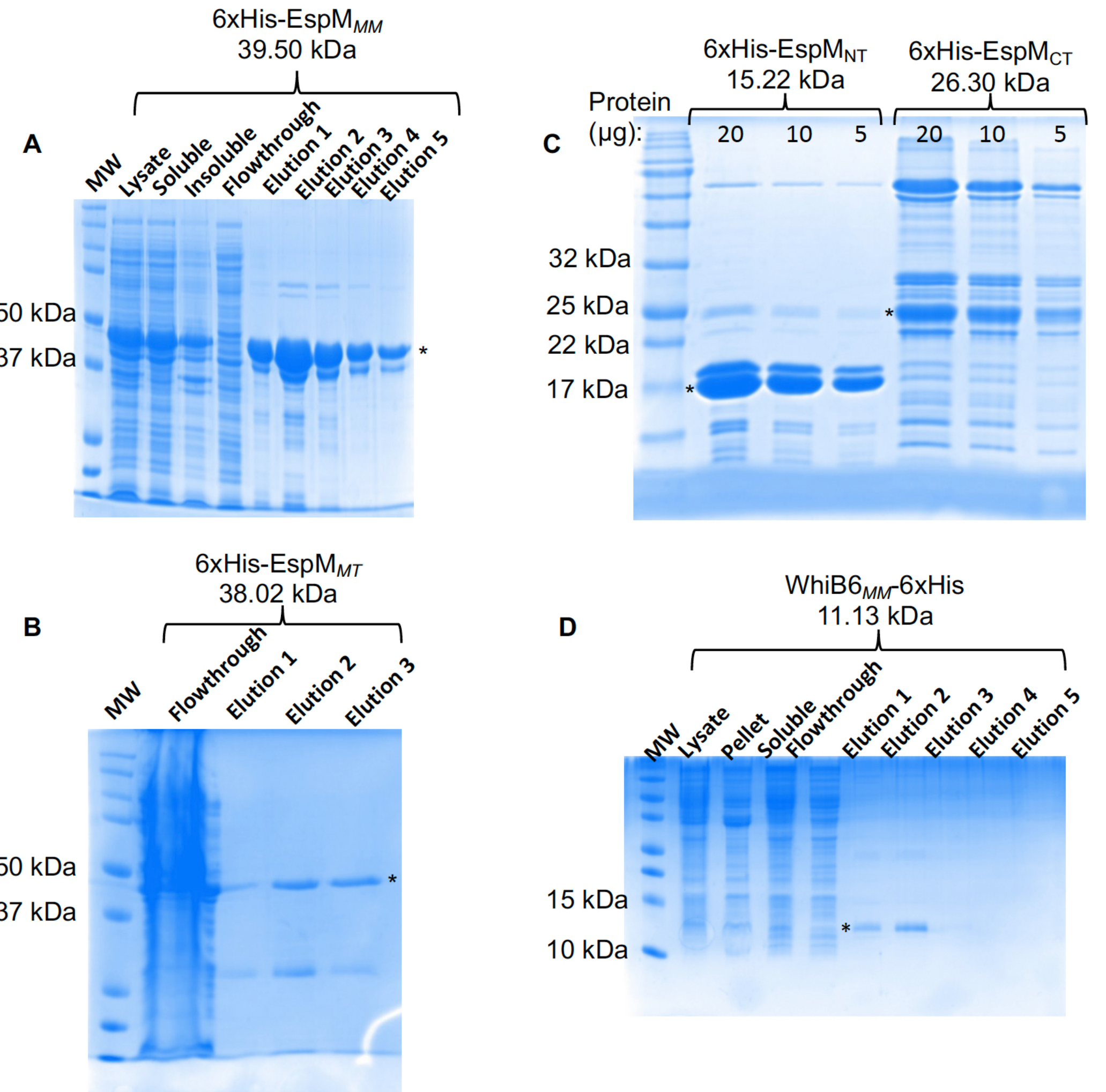

Supplement: FIG S2 [file mBio.02807-19-sf002.pdf]

Figure S4

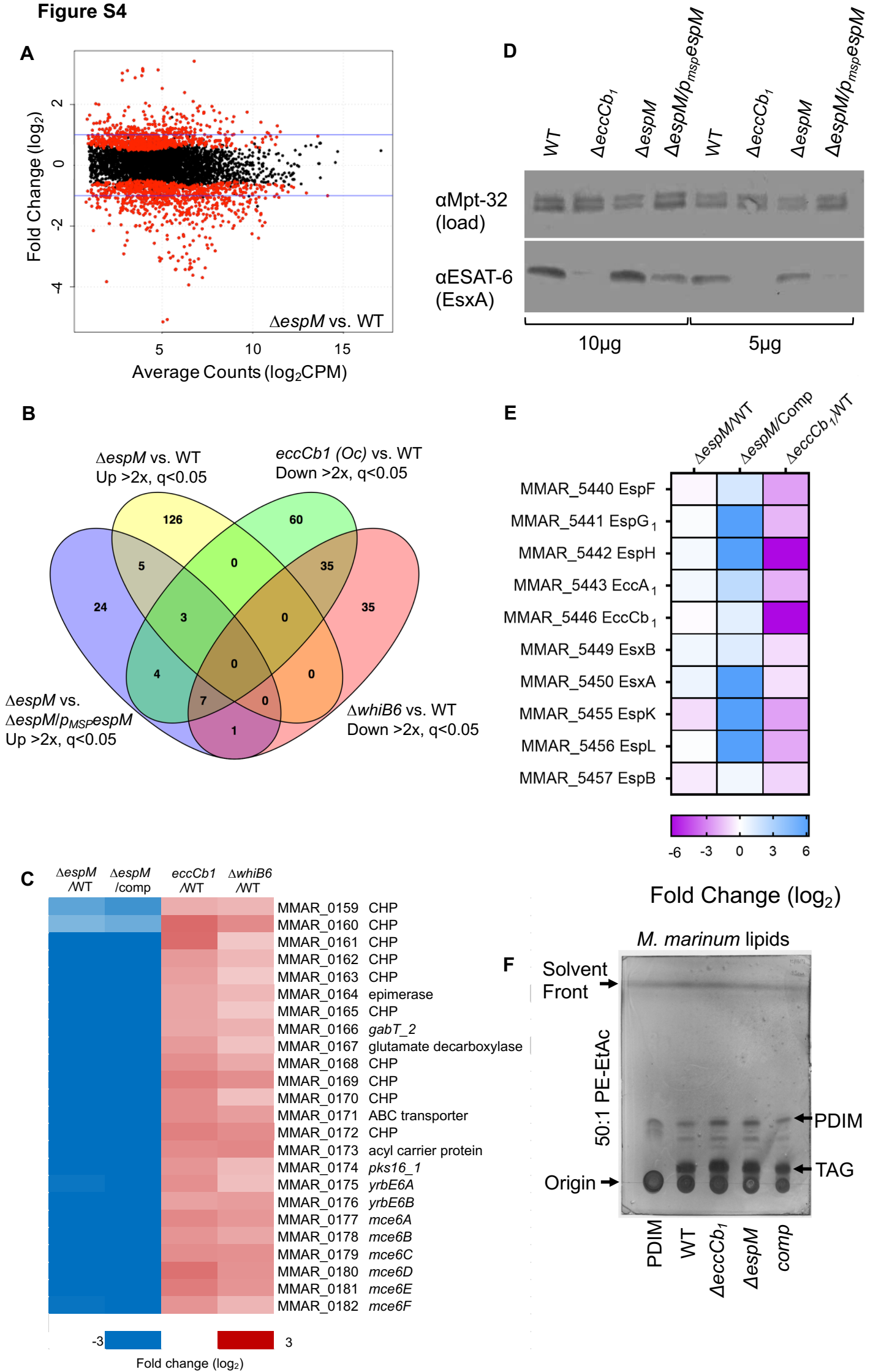

Supplement: FIG S4 [file mBio.02807-19-sf004.pdf]

## Figure S5

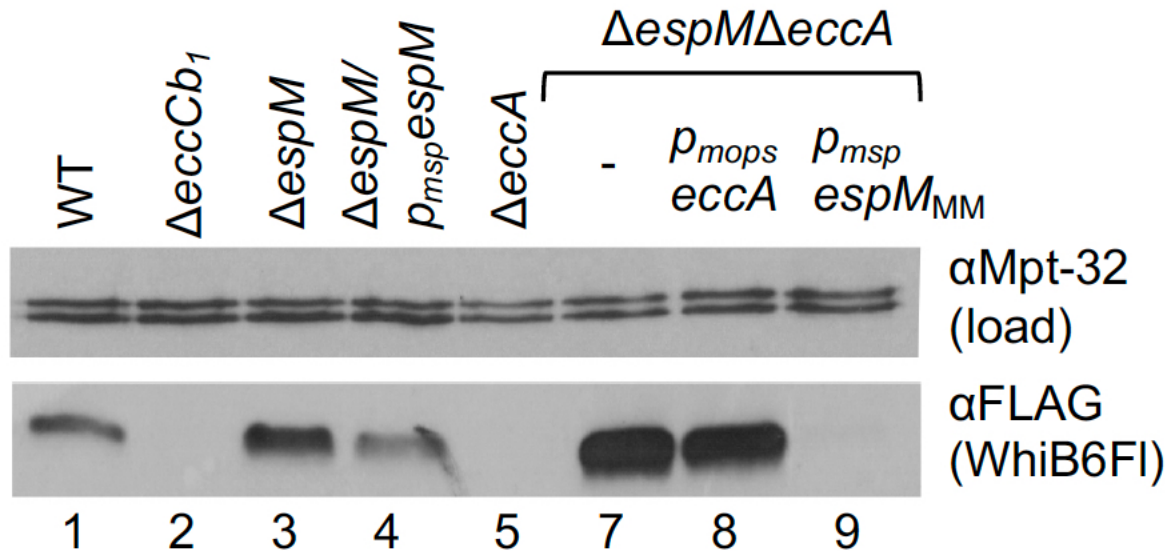

Supplement: FIG S5 [file mBio.02807-19-sf005.pdf]

Figure S6

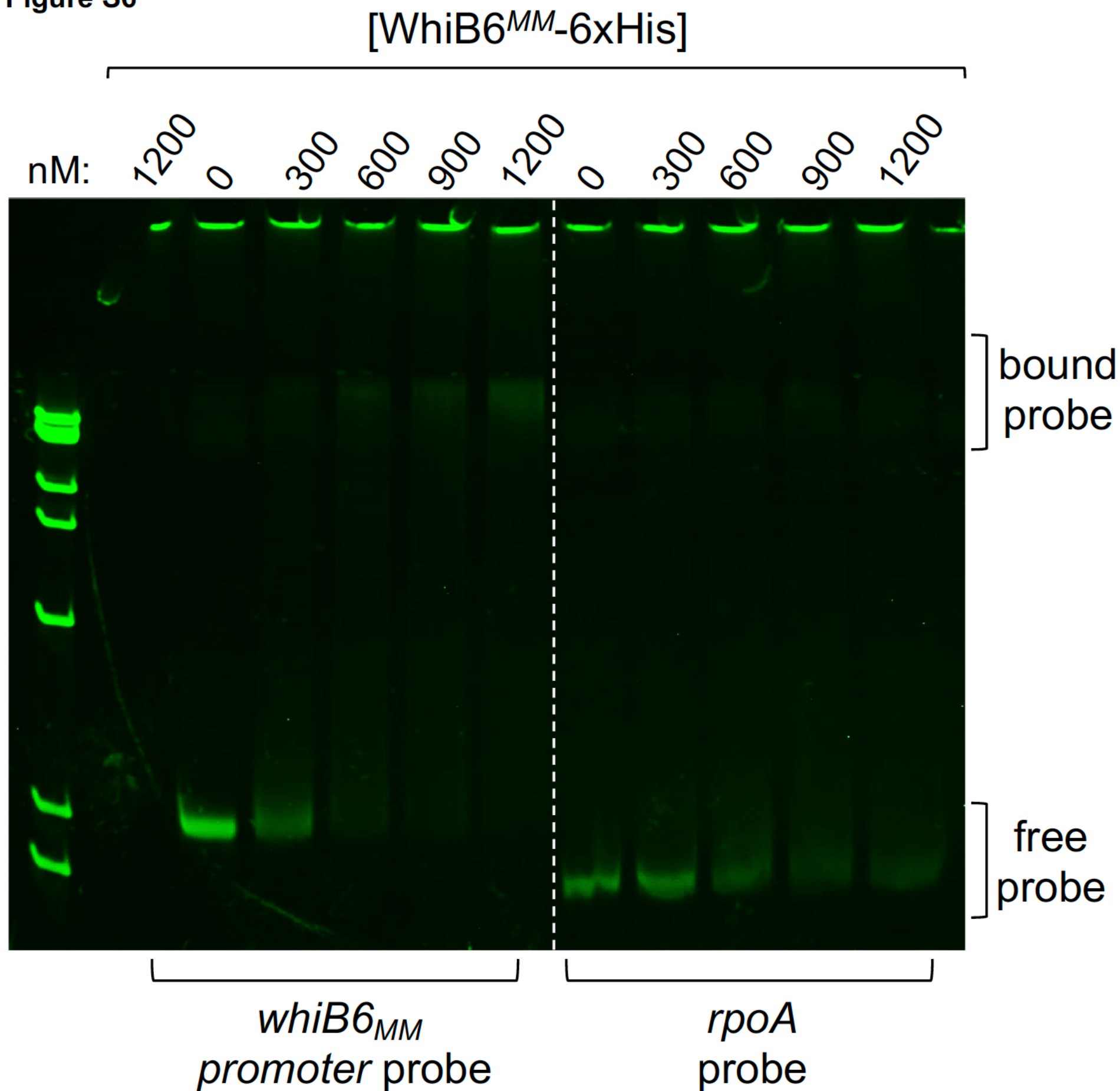

Supplement: FIG S6 [file mBio.02807-19-sf006.pdf]
