# Supplementary material for: EspM Is a Conserved Transcription Factor That Regulates Gene Expression in Response to the ESX-1 System
Source: mBio. 2020 Feb 4;11(1):e02807-19. doi: 10.1128/mBio.02807-19 (PMC7002343; doi:10.1128/mBio.02807-19)
Supplement: TEXT S1 [file mBio.02807-19-s0001.pdf]

Supporting Materials and Methods:

Maintenance of bacterial strains. *M. marinum* strains were maintained in Middlebrook 7H9 defined broth (Sigma-Aldrich, St Louis MO) supplemented with 0.5% glycerol and 0.1% Tween-80 (Fisher Scientific, Pittsburgh PA) or on Middlebrook 7H11 agar (Sigma-Aldrich) plates supplemented with 0.5% glycerol and 0.5% glucose. *M. marinum* was maintained at 30°C. When necessary, agar plates and broth were supplemented with 20 µg/mL kanamycin (IBI Scientific, Dubuque, IA) / 50 µg/mL hygromycin (Sigma-Aldrich). Strains with integrating plasmids were grown in the absence of antibiotics. *E. coli* strains were maintained in in LB (Luria-Bertani) media (VWR) with 50 µg/mL kanamycin, 200 µg/mL hygromycin or 200 µg/mL ampicillin (Thermo Fisher, Waltham, MA) where appropriate. All cloning and plasmid propagation were done in strain DH5α (New England Biolabs, Ipswich, MA) and all protein expression was done in strain BL21 Star (DE3) (Thermo Fisher).

Nomenclature. The nomenclature in this study adheres to Bitter et al. (W. Bitter et al. PLoS Pathog. 5(10):e1000507., 2009. doi: 10.1371/journal.ppat.1000507.). ESX-1 conserved components are designated Ecc. ESX-1 associated proteins are designated Esp. Subscript 1 indicates genes or proteins associated with the ESX-1 system. Subscripts are also used to indicate species. Proteins or genes indicated with *MM* are from *M. marinum* M, *MT* are from *M. tuberculosis* Erdman, and *MS* are from *M. smegmatis* mc$_2$155.

DNA Affinity Chromatography. DNA affinity chromatography was performed as described in Chaparian et al. (R.R. Chaparian et al, Mol Microbiol. 2016 Sep;101(5):823-40, 2016. doi: 10.1111/mmi.13425.). The *whiB6-espM* and *rpoA* biotinylated probes were amplified from *M. marinum* M genomic DNA, which was prepared as described (R. E. Bosserman *et al*. *Proc Natl Acad Sci U S A* 114(50):E10772-E10781, 2017 doi: 10.1073/pnas.1710167114.). Biotinylated probes were generated

as follows**.** The 1 kb region upstream of the start of the *whiB6* open reading frame (40 bp into *whiB6* to 486 bp into *MMAR_5438;* 6577326-6578305 bps in the *M. marinum* M genome) was amplified by PCR from *M. marinum* M genomic DNA using the OKS18/19 oligonucleotide primers (Table S2). The *rpoA* probe was amplified by PCR using the RpoAFwd/Rev oligonucleotide primers. The resulting biotinylated probes were concentrated to 200 ng/µL. 100-150 µL of each probe was coupled to Streptavadin M-280 Dynabeads (Thermo Fisher) Conjugation of probes to the beads was performed as follows. The beads were incubated with the probe for 30 minutes on a nutator at room temperature. DNA depletion of the flow through was confirmed A_{260} spectrophotometry. The charged beads were washed three times with 500 µL of 2x BW buffer (10 mM Tris-Cl, pH 7.5, 1 mM EDTA, 2 M NaCl), three times with 500 µL of 1x TE buffer (10 mM Tris-HCl, pH 8.0, 1 mM EDTA), and twice with 700 µL of BS-THES buffer (22 mM Tris-HCl, pH 7.5, 4.4 mM EDTA, 8.9% (w/v) sucrose, 62 nM NaCl 0.3% protease inhibitor cocktail (PIC) (Sigma), 0.04% PMSF, 10 mM, HEPES, 5 mM $CaCl_2$, 50 mM KCl, and 12% glycerol). The second wash with BS-THES included 10 mg/mL salmon sperm DNA. 50 mL cultures of *M. marinum* were grown in 7H9 broth to an OD_{600} of ~2.5. Lysates were generated and protein concentration was determined as described (R. E. Bosserman *et al*. *Proc Natl Acad Sci U S A* 114(50):E10772-E10781, 2017 doi: 10.1073/pnas.1710167114.). Clarified lysate (600-1000 µg) was incubated with the charged Dynabeads, the beads were immobilized and washed. Following lysate preparation, 200 µL of the clarified lysate (600-1000 µg) was incubated at 4°C overnight with the Dynabeads charged with each probe and 800 µL of BS-THES buffer containing 10 mg/mL of salmon sperm DNA. After overnight incubation, the beads were immobilized and washed five times with 500 µL of BS-THES buffer, with 10 mg/mL salmon sperm DNA. Proteins were then eluted three times with 60 µL of 1 M NaCl in 25 mM Tris-HCl. The pooled elutions were analyzed using MS.

Proteomics. For the DNA pull down presented in Figure 1**,** Proteins were then eluted and analyzed using quantitative nano UHPLC-MS/MS proteomics. Briefly, protein elutions were dried down by vacuum centrifugation (Genvac, Warminster, PA), washed with 200 μL ice-cold acetone and allowed to dry. Proteins were denatured in 5% SDS, reduced with DTT, alkylated with iodoacetamide (Sigma-Aldrich) and prepared for trypsin digestion and MS-MS/MS analysis using S-traps (A. Zougman, P. J. Selby, and R. E. Banks, *Proteomics* 14:1006-1000, 2014.doi: 10.1002/pmic.201300553.). Samples were acidified using 1/10 v/v 12% H_3PO_4 and flocculated by 7-volume addition of 95/5 MeOH:50mM ammonium bicarbonate (ABC) (v/v). Protein aggregates were filtered on a commercial S-trap (Protifi, Huntington, NY), washed with 95/5 MeOH:ABC., hydrated in 1μg trypsin (5ng/μL) in 50mM ABC and digested for 8h prior to elution, concentration and desalting with C18 as previously described in R. E. Bosserman *et al*. *Proc Natl Acad Sci U S A* 114(50):E10772-E10781, 2017 doi: 10.1073/pnas.1710167114 and R. E. Bosserman, et al. J Bacteriol 201(14). 2019. pii: e00760-18. doi: 10.1128/JB.00760-18. Mass spectrometry was performed essentially as in R. E. Bosserman *et al*. *Proc Natl Acad Sci U S A* 114(50):E10772-E10781, 2017 doi: 10.1073/pnas.1710167114 and R. E. Bosserman, et al. J Bacteriol 201(14). 2019. pii: e00760-18. doi: 10.1128/JB.00760-18 and R. M. Robinson *et al.*,. Leukemia 33(4):1011-1022. 2019. doi: 10.1038/s41375-018-0263-1) with biological replication and technical duplication. RAW LC-MS/MS files were searched using MaxQuant v1.6.2.3 against the *M. marinum* database from Uniprot 5,425 entries (05-2019) with common contaminants. Proteins were cutoff at a global FDR of 1% and quantification was performed using Max LFQ with default parameters and normalized intensities were reported (J. Cox et al., Mol Cell Proteomics 13(9):2513-26. 2014. doi: 10.1074/mcp.M113.031591). 352,197, and 139 proteins were identified in the biological replicates at a 1% FDR respectively. Data from a representative replicate (E.A. Williams et al. Infect Immun., 85(2). pii: e00653-16, 2017. doi: 10.1128/IAI.00653-16) were used for figures. Protein intensities were normalized to the mean intensity of the non-specific nucleic acid binding protein

HupB. Fold enrichment was measured as the ratio of normalized intensity with *whiB6-espM* probe *vs* normalized intensity with the *rpoA* DNA probe (P. A. Champion, et al. Mol Microbiol 73: 950-962, 2009. doi: 10.1111/j.1365-2958.2009.06821.x.). In cases where a protein was quantifiable or measured in the *rpoA* control (e.g. EspM with the *rpoA* probe) the fold-enrichment was thresholded to the ratio of the enriched protein to the mean of the lowest intensity protein measured in the samples, and then capped at the approximate dynamic range of the experiment (2^6). Error was determined by propagation of the error of the %CV for HupB across the samples. Normalized %CV for HupB across all replicates was ±4%.

For the quantitative proteomics in Figure S4 panel E, Proteins were prepared for mass spectrometry based quantitative proteomics essentially as described in R. E. Bosserman, et al. J Bacteriol 201(14). 2019. pii: e00760-18. doi: 10.1128/JB.00760-18 and R. E. Bosserman *et al*. *Proc Natl Acad Sci U S A* 114(50):E10772-E10781, 2017 doi: 10.1073/pnas.1710167114. Briefly, 50µg of protein from whole cell lysates of the WT (*whiB6-FL),* Δ*espM,* Δ*espM*/p_{msp}*espM* and the Δ*eccCb1 M. marinum* strains were reduced, alkylated and digested with trypsin using S traps (Protifi, NY). Nano uHPLC MS/MS was acquired as described in R. E. Bosserman, et al. J Bacteriol 201(14). 2019. pii: e00760-18. doi: 10.1128/JB.00760-18 on a QE-HF (Themo, San Jose, CA) mass spectrometer running in DDA TOP 15 mode. MS-MS/MS searching was done as described above. Data reduction and (Label-Free) LFQ-based quantification are in Table S1E and S1F. Protein quantification was averaged over technical duplicate and biological replicate and normalized per-biological replicate. Error per replicate measurement was taken as %CV. Fold change was limited to +/- 6.2 (Log_2) to describe proteins which were absent in one sample but confidently quantified in another (e.g. $EccCb_1$ in WT *vs* Δ$eccCb_1$) and reflects an appropriate dynamic range of LFQ-Proteomics.

Electrophoretic Mobility Shift Assays. The *whiB6/MMAR_5438* intergenic region used for the probe in Figure 1 (6577396-6577960 bps in the *M. marinum* M genome; 30 bp upstream of the *whiB6* ORF to 141 bp into *MMAR_5438*) was amplified from *M. marinum* M genomic DNA using PCR with the oligonucleotide primers OKS77/OKS88. The *rpoA* coding region was amplified by PCR using the RpoAFwd/Rev oligonucleotide primers. ~70 ng of each probe was incubated with increasing concentrations of purified 6xHis-EspM in 0.5x binding buffer (10 mM Tris-HCl (pH 8.0 RT), 40 mM KCl, 2.5 mM DTT, 0.25% Tween-20, and 0.02 mg/mL salmon sperm DNA) and incubated for 20-25 minutes at 4°C. The probes were resolved on a non-denaturing 2-4% polyacrylamide TAE gel, supplemented with 0.5% glycerol. The gel was precleared for 1-3 hours on ice at 100 V in a 0.37x TAE solution. The probes were run for 3 hours at 100 V using Bio-Rad mini-PROTEAN tetra vertical electrophoresis system. A mixture of xylene cyanol, bromophenol blue and orange G dyes were run alongside to monitor migration. Probes were visualized by staining with 1/50000x SYBR green stain (Thermo Fisher) in 1x TAE. The gel was briefly washed twice in water and imaged on a Gel Doc EZ Imager (Bio-Rad, Hercules, CA) with ImageLab software (Bio-Rad).

EMSAs determining if 6xHis-WhiB6 bound the *whiB6* promoter were performed in the exact same way with the exact same probes. The EMSAs were performed in 0.5x binding buffer (40 mM HEPES-HCl (pH 7.5), 50 mM KCl, 2 mM $MgCl_2$, and 0.01 mM EDTA). The probes were resolved and imaged as stated previously.

Expression and purification of 6xHis-EspM proteins. The *MMAR_5438* ORF was amplified from M strain *M. marinum* genomic DNA using Phusion polymerase (NEB) and the primers OMF624 and OMF625 (IDT). Cloning vector pET15 vector (Novagen) was prepared for FastCloning (C. Li et al., BMC Biotechnol **11:**92, 2011. .doi: 10.1186/1472-6750-11-92.) by PCR amplification with primers OMF512 and OMF513 followed by treatment with DpnI (NEB). Vector and insert were mixed and

introduced into chemically competent DH5α *E. coli* (Thermo Fisher), plated on LB (VWR) agar with 200 μg/mL ampicillin (Thermo Fisher) and incubated at 37°C. The resultant colonies were grown overnight at 37°C in LB ampicillin broth followed by plasmid miniprep (Bioneer). Plasmids were evaluated by restriction digest with NdeI and SpeI (NEB).

Truncation expression constructs were created by PCR amplification from M strain *M. marinum* genomic DNA with the primers OM624 and OMF626 (N-terminal domain containing FHA), OMF625 and OMF627 (C-terminal domain containing HTH) and introduced into pET15 as above.

espM$_{MT}$ from *M. tuberculosis* strain Erdman (ERDMAN_4236) was initially amplified using PCR from *M. tuberculosis* Erdman gDNA with primers ERD-4236-F and ERD-4236-R followed by FastCloning into the bacterial 2-hybrid vector pKNT25 (Euromedex) prepared by amplification with the primers V-KT25-F and V-KT25-R. This gene was excised by restriction digest with NdeI and SpeI and ligated into pET15 vector prepared by cutting pET15-mCherry with NdeI and SpeI followed by dephosphorylation with Antarctic Phosphatase (NEB). Plasmids were evaluated by restriction digest with NdeI and SpeI and agarose gel electrophoresis. pET15-mCherry vector was created by PCR amplifying mCherry from pET-mCherry-LIC cloning vector, a gift from Scott Gradia (Addgene# 29769) with primers OMF514 and OMF515 and introduction using FastCloning into pET15 as above.

whiB6 from *M. marinum* was amplified from wild-type gDNA using the primers OMF607 and OMF608. PCR product was cloned into the pET29 vector (Novagen) prepared for FastCloning by amplification with primers OMF313 and OF606.

The resulting constructs were introduced into chemically competent BL21(DE3) Star *E. coli* (Invitrogen). 5 mL starter cultures grown in LB with antibiotics were used to inoculate 500mL of Auto-Induction Media (F. W. Studier, Protein Expr Purif 41: 207-234, 2005.) with either 200 μg/mL ampicillin (pET15 constructs) or 50 μg/mL kanamycin in 2 L baffled flasks (Thermo Fisher). Shaking cultures were grown at 37°C for 4 hours then shifted to 30°C for 18 hours. Bacterial cells were harvested by

centrifugation at 4000 RPM for 15 minutes in 400 mL bottles in an Eppendorf 5810 centrifuge fitted with a A-4-81 rotor. Pellet was washed by resuspension in 40 mL of PBS and centrifugation for 15 minutes in 50 mL conical tubes. The washed cell pellet was stored at -80°C until purification.

The pelleted cells were thawed and resuspended in lysis buffer [300 mM KCl (Thermo Fisher), 2mM $MgCl_2$ (Thermo Fisher), 20 mM HEPES (Thermo Fisher) pH 7.0, 10% glycerol (Macron), 0.5% CHAPS (Sigma), 4 µg/mL lysozyme (Sigma), 60 µg/mL PMSF(Roche)] and lysed using a microtip sonicator (Branson Ultrasonics) using 2 second pulses at 25% amplitude. 2500 units of universal nuclease (Pierce) was added to the lysate and incubated at room temperature for 30 minutes with gentle agitation. The treated lysate was clarified by centrifugation for 30 minutes at 7800 RPM in an Eppendorf 5430 with an F-35-6-30 rotor. Clarified lysate was mixed with 10 ml of Ni-NTA resin (GE) in a 50% slurry with binding buffer [300 mM KCl, 2mM $MgCl_2$, 20 mM HEPES pH 7.0, 40 mM imidazole (Sigma), 10% glycerol]. Resin-lysate mixture was incubated for 3 hours at 4°C with gentle agitation on a nutator. Resin was washed three times batchwise with 40 mL (8 CV) of binding buffer. Washed resin was loaded into an empty PD-10 gravity column (GE) and washed with an additional 20 mL of cold binding buffer. Protein was eluted in 5 mL (1 CV) fractions with elution buffer (300 mM KCl, 2mM $MgCl_2$, 20 mM HEPES pH 7.0, 300 mM imidazole, 10% glycerol). Elutions were evaluated by dotting on filter paper and staining with Coomassie blue stain diluted 1:4 in water followed by destaining in running deionized water. Normalized samples of lysate, insoluble, soluble, flow-through and elutions were evaluated by SDS-PAGE on 12% tris-glycine gels followed by Coomassie staining. Elutions were pooled and concentrated by ultrafiltration in a 3000 MWCO Amicon (Millipore), aliquoted in PCR tubes and flash-frozen in liquid nitrogen and stored at -80°C. Final products were assayed by BCA assay (Pierce) with His6-EspM at 7.6 mg/mL, His6-EspM$_{NT}$ at 26.1 mg/mL, His6-EspM$_{CT}$ at 2.3 mg/mL, and His6-WhiB6 at 2.0 mg/mL. Purified proteins were confirmed using LC/MS/MS.

Denaturing purification and refolding of His6-EspM$_{MT}$. His6-EspM$_{TB}$ was expressed from the pET15 construct and the cells were lysed as above. The insoluble fraction was dissolved in freshly prepared binding buffer supplemented with 8M urea (Sigma). The dissolved pellet was clarified by centrifugation and mixed with buffer equilibrated Ni-NTA resin and incubated overnight at 4°C with gentle agitation on a nutator. Resin was washed three times batchwise with 40 mL (8 CV) of urea binding buffer. The protein was refolded by elution in urea-free elution buffer. Elutions were evaluated for purity by SDS-PAGE and Coomassie staining and pure elutions were pooled, concentrated, aliquoted and frozen as above. Final concentration was determined to be 0.48 mg/mL by BCA assay.

Generation of the *espM* deletion and overexpression strains. Primers to amplify the flanking regions of *MMAR_5438* (*espM*) were designed using the May 2017 version of the *M. marinum* str. M genome (GID 183980035) obtained from NCBI with the *MMAR_5438* ORF between 6577820 and 6578908 bp. Note that the 2017 annotation results in a protein that is lacking the first 22AA of the protein predicted in Mycobrowser [MLVSGTRALPPASVAASEESTY, (A. Kapopoulou, J. M. Lew, and S. T. Cole, Tuberculosis (Edinb) 91: 8-13, 2011.doi: 10.1016/j.tube.2010.09.006)]. Upstream and downstream sequences were amplified from genomic DNA prepared from the *whiB6*-Fl strain (R. E. Bosserman *et al*. *Proc Natl Acad Sci U S A* 114(50):E10772-E10781, 2017 doi: 10.1073/pnas.1710167114.) using the primer pairs OMF615, OMF616 and OMF617, OMF618. PCR products were introduced into the p2NIL vector [Addgene plasmid #20188, a gift from Tanya Parish (T. Parish and N.G.Stoker, Microbiology, 146 (Pt 8):1969-75, 2000, https://doi.org/10.1099/00221287-146-8-1969)] by three part fast-cloning as previously described (R. E. Bosserman *et al*. *Proc Natl Acad Sci U S A* 114(50):E10772-E10781, 2017 doi: 10.1073/pnas.1710167114.). Constructs were digested with PacI (NEB) and dephosphorylated using Antarctic Phosphatase (NEB) followed by heat treatment at 65°C. pGOAL19 [Addgene plasmid #20190, a gift from Tanya Parish, (T. Parish and N.G.Stoker,

Microbiology, 146 (Pt 8):1969-75, 2000, https://doi.org/10.1099/00221287-146-8-1969)] was digested with PacI followed by 65°C heat treatment. The GOAL cassette was ligated into p2NIL_5438KO construct using T4 DNA ligase (NEB) at 4°C overnight. Ligations were introduced into DH5α *E. coli* and plated on LB agar with 50 µg/mL kanamycin (IBI) and 60 µg/mL X-Gal (RPI), blue colonies were cultured in LB kanamycin broth and plasmids were purified by miniprep (Bioneer). Plasmids were checked by restriction double digest with PacI and AflII followed by agarose gel electrophoresis.

Plasmids were quantified by A260 on nanodrop (Thermo Fisher) and 3 µg of plasmid was transferred to a sterile microfuge tube and irradiated with 0.1 J/cm2 UV light in a CL-1000 ultraviolet crosslinker (UVP) followed by electroporation into 500 µl or electrocompetent *whiB6-Fl M. marinum* using a GenePulser XCell (BioRad). Electrocompetent cells were prepared by growth in 7H9 broth (Sigma) with 0.1% tween 80 (Amresco) to mid-log phase followed by the addition of sterile glycine (Sigma) to 1.5% w/v (R. A. P. Goude, Methods in Molecular Biology, T. P. a. D. M. Roberts, Ed. (Springer, 2015), chap. Electroporation in Mycobacteria, pp. 117-130). 12 hours before harvest. Harvested cells were washed three times in sterile 10% glycerol with 0.1% tween 80, aliquoted into sterile tubes and stored at -80°C. Transformations were promptly transferred to 15 mL conical tubes with 2.5 mL of 7H9 +0.1% tween 80 broth. Transformations were incubated overnight at 30°C. Recovered transformations were briefly vortexed and pelleted by centrifugation, media was decanted and the pellet was resuspended in 100 µL of media and plated on 7H11 agar (Sigma) supplemented with OADC (BD), 20 µg/mL kanamycin and 60 µg/mL X-Gal. Plates were inverted and incubated for 14 days at 30°C.

Blue colonies were picked and grown in 7H9 broth followed by confirmation of the genotype using PCR with the primers OMF619, OMF622 and OMF619, OMF620. Confirmed merodiploids were plated on 7H11 agar supplemented with OADC, 60 µg/mL X-Gal and 2% sucrose (Macron) and grown for 14 days at 30°C. White colonies were picked and cultured for 5 days in 3 mL of 7H9 broth with 0.1%

tween 80. Cultures were checked for deletion using a rapid genotyping process. Briefly, 250 µL of cultures were transferred to screw-cap tubes and cells were collected by centrifugation, media was decanted and 0.1 mm zirconia disruption beads (RPI) and 500 µL of TE buffer were added. Pellets were lysed by three 30 second pulses on a mini bead beater (Biospec Products) followed by clarification by 5-minute centrifugation. Clarified lysate were diluted 1:10 in sterile water and 1 µL of diluted template was used in 10 µL PCR reactions with GC buffer and 4% DMSO using the primers OMF619 and OMF620. PCR products were run on 0.8% TAE agarose gels stained with ethidium bromide (VWR) and imaged using a Gel Doc EZ imager (BioRad) and Image Lab software (BioRad). Amplicons of 1364 bp and 335 bp were interpreted as wild type or knockouts, respectively.

To create constructs for overexpression of *espM* in *M. marinum,* individual genes (*MMAR_5348*, *MSMEG_0052*, *ERDMAN_4236*) were cut out of their respective pET15 constructs using NdeI and SpeI restriction enzymes and ligated into pMSP vector prepared by NdeI SpeI digesting pMSP-mOrange. pET15-MSMEG_0052 was made by amplifying *MSMEG_0052* from *M. smegmatis* mc_2155 gDNA using primers OMF631, OMF632 and Phusion polymerase followed by FastCloning into pET15 prepared as above. p_{msp}mOrange vector was derived from a p_{mop}-mOrange construct initially made by FastCloning mOrange amplified from pET-mOrange-LIC cloning vector, a gift from Scott Gradia (Addgene #29770) with primers OMF047, OMF048 into pMOP vector prepared with primers OMF004, OMF042. pMOP-mOrange was prepared for FastCloning by amplification with OMF074, OMF083 and the MSP promoter was amplified from pR2Hyg-dsRed with OMF072, OMF082.

pMSP overexpression constructs were introduced into the *whiB6-Fl* Δ*espM* strain by electroporation of 500 ng of purified plasmid into 500 µL of electrocompetent cells prepared as above. Transformations were promptly transferred to 15 mL conical tubes and mixed with 2.5 mL of 7H9 broth supplemented with 0.1% tween 80 and recovered overnight at 30°C on a wheel nutator. Recovered cultures were briefly vortexed and pelleted by centrifugation. Pelleted cells were resuspended in 100

µL of media and plated on 7H11 agar supplemented with 0.5% glycerol, 0.5% glucose (Sigma) and 50 µg/mL hygromycin followed by incubation at 30°C for 14-20 days. Individual colonies were struck for isolation on 7H11 plates and incubated for at 30°C for 10-14 days.

Generation of the *espM ESX-1* double knockout strains. Plasmid p2NIL-Δ*eccA*$_1$ GOAL was constructed by amplifying the flanking regions of *eccA*$_1$ using the primers OMF180, OMF181 and OMF182, OMF183 followed by fast cloning into p2NIL vector. Vector was prepared by PCR amplification with primers OMF096 and OMF097 followed by DpnI (NEB) treatment. Cloning reactions were introduced into DH5α *E. coli*.

Plasmids p2NIL-Δ*eccA*$_1$ GOAL, p2NIL-Δ*eccCb*$_1$ GOAL (E.A. Williams et al. Infect Immun., 85(2). pii: e00653-16, 2017. doi: 10.1128/IAI.00653-16) and p2NIL-Δ*eccCa*$_1$ GOAL, p2NIL-Δ*eccB*$_1$ GOAL, p2NIL-Δ*eccD*$_1$ GOAL and p2NIL-Δ*eccE*$_1$ GOAL (R. E. Bosserman *et al*. *Proc Natl Acad Sci U S A* 114(50):E10772-E10781, 2017 doi: 10.1073/pnas.1710167114.) were introduced into electrocompetent *whiB6*-FI Δ*espM M. marinum* and plated on 7H11 agar supplemented with OADC, 20 µg/mL kanamycin and 60 µg/mL X-Gal. Blue colonies were picked at 14 days, grown 5 days in 7H9 + 0.1% tween 80 broth and plated on 7H11 agar supplemented with OADC, 60 µg/mL X-Gal and 2% sucrose and grown for 14 days at 30°C. White colonies were picked and grown in broth culture for 5 days followed by PCR genotyping with Taq polymerase (NEB). Deletions of *eccA*$_1$ were confirmed using the primers OMF184 and OMF185 with amplicons of 2216 and 557 bp were interpreted as wild type and Δ*eccA*$_1$, respectively. Deletions of the *eccB*$_1$ gene were checked with primers OMF190 and OMF191 with amplicons of 2907 and 500 bp being interpreted as wild type and Δ*eccB*$_1$, respectively. Deletions of the *eccCa*$_1$ gene were checked with primers OMF196 and OMF197 with amplicons of 2908 and 712 bp being interpreted as wild type and Δ*eccCa*$_1$, respectively.

Deletions of *eccCb*$_1$ were confirmed using the primers orb72 and orb73 with amplicons of 3639 and 1878 bp being interpreted as wild type and Δ*eccCb*$_1$, respectively. Deletions of the *eccD*$_1$ gene were confirmed with primers OMF202 and OMF203 with amplicons of 1959 and 567 bp being interpreted as wild type and Δ*eccD*$_1$, respectively. Deletions of the *eccE*$_1$ gene were confirmed with primers OMF208 and OMF209 with amplicons of 1747 and 421 bp being interpreted as wild type and Δ*eccE*$_1$, respectively.

The *eccA*$_1$ complementation construct was constructed by amplifying the *eccA*$_1$ gene from wild type *M. marinum* M gDNA using Phusion polymerase and primers OMF645, OMF646 followed by fast cloning into pMOP vector prepared using primers OMF057 and OMF630. The resulting p$_{MOP}$*eccA*$_1$ construct was checked for insert by restriction digest with NdeI and SpeI. 500ng of construct was introduced into the *whi66-Fl*Δ*eccA*$_1$Δ*espM cells* prepared as above followed by plating on 7H11 with 50 µg/mL hygromycin.

Generation of Δ*whiB6* Δ*espM* Double Knockout Strain. The construct for deleting *whiB6* in an Δ*espM* background was generated by amplifying *ΔwhiB6* gDNA with primers OMF615, OMF616 and OMF617, OMF618 and fast cloning into the p2NIL vector. The resulting plasmid was confirmed by EcoRI and NcoI (NEB) restriction digest. The GOAL cassette was ligated into confirmed constructs as above and confirmed by AflII PacI (NEB) restriction digest. The resulting p2NIL-Δ*whiB6*-Δ*espM*-GOAL plasmid was UV irradiated and introduced into *whiB6-Fl*Δ*espM M. marinum* as above. Blue colonies were picked and grown in 7H9 followed by plating on sucrose counterselection media as above. The resolved white colonies were picked and grown in 7H9 followed by genotyping with Taq polymerase and the primers OMF620 and OMF709. A product of 1113 bp was interpreted as the *whib6-Fl*Δ*espM* parental while a product of 831bp was interpreted as the Δ*whiB6*Δ*espM* strain.

Generation of *whiB6*::*lacZ*+ Strains. Construct for introducing *lacZ* behind the *whiB6* promoter was created by amplifying the existing construct p2NIL-ΔwhiB6 [an intermediate in the construction of the *whiB6* knockout construct, (R. E. Bosserman *et al*. *Proc Natl Acad Sci U S A* 114(50):E10772-E10781, 2017 doi: 10.1073/pnas.1710167114.)] with the primer pairs OMF049, ors221 and OMF050, OMF351. *lacZ* was amplified from pGOAL19 using the primers OMF352 and OMF353. PCR products were DpnI treated, introduced into DH5α *E. coli* and plated on LB agar with 50 μg/mL kanamycin, 60 μg/mL X-Gal. The confirmed plasmids were UV treated and introduced into Δ*whiB6* and Δ*whiB6* Δ$eccCb_1$ strains as above. Blue merodiploid colonies were picked and plated on sucrose as above. Blue sucrose resistant colonies were evaluated by PCR using the primers orb14 and OMF174 with a 3283 bp product being interpreted as a successful integration of *lacZ*. The plasmid p_{msp}*espM* was introduced to the *whiB6*::*lacZ* strain by electroporation.

Construction of *lacZ*+ reporter plasmids. An integrating *whiB6::lacZ* reporter plasmid was constructed by amplifying the *lacZ* reporter and the 454 bps upstream from Δ$eccCb_1$ *whiB6*::*lacZ* gDNA with primers OMF355, OMF356 followed by fast cloning into pMV306Hyg vector amplified with primers ORS107, ORS112 followed by DpnI treatment. Constructs were checked by digesting with a combination of EcoRI, EcoRV and HindIII (NEB).

An integrating *espM::lacZ* reporter plasmid was constructed by PCR amplifying sequence between 545 bp upstream of *espM* (including the first 100nt of the *whiB6* ORF) and base 3 of the *espM* ORF (2017 annotation) using primers OMF701, OMF733. *lacZ* was amplified from pGOAL19 using OMF353 and OMF734. pMV306Hyg vector prepared by PCR amplification with OMF074, ORS107 followed by DpnI treatment. Upstream sequence and *lacZ* were cloned into pMV306Hyg by three-part fast cloning and checked by EcoRI plus EcoRV double digest.

Protein Alignments. All protein alignments were performed by using Clustal Omega (F. Sievers et al., Mol Syst Biol 7:539, 2011.doi: 10.1038/msb.2011.75). and were visualized using BoxShade (https://sourceforge.net/projects/boxshade/).

Transcriptional Profiling. *M. marinum* was cultured in 5 mL of 7H9 and 0.1% Tween-80 for two days, then moved to 25 mL 7H9 and 0.1% Tween-80 for two days. All strains were then incubated in Sauton's media 0.01% Tween-80 at an OD of 0.8 for 48 hours. The cultures were collected by centrifugation, frozen, and then lysed using a bead beater. Total RNA was prepared from the pellets using the Qiagen RNeasy kit (Qiagen, Germantown, MD) (R. E. Bosserman *et al*. *Proc Natl Acad Sci U S A* 114(50):E10772-E10781, 2017 doi: 10.1073/pnas.1710167114.).

Western blot analysis: Western blot analysis to detect ESX-1 protein secretion, or WhiB6-Fl levels were performed as follows. All antibodies were diluted in 5% nonfat dry milk in PBS 0.1% Tween 20. RNA polymerase subunit β (RpoB; 1:10,000; ab12087; Abcam), ESAT-6 (EsxA; 1:5,000; HYB 076-08-02; Thermo Fisher), and Monoclonal Anti- FLAG M2 (1:5,000; Sigma-Aldrich). The following reagents were obtained through BEI Resources, NIAID, NIH: Polyclonal Anti-*Mycobacterium tuberculosis* CFP10 (Gene Rv3874) (antiserum, rabbit), NR-13801 and Polyclonal Anti-Mycobacterium tuberculosis Mpt32 (Gene Rv1860) (antiserum, rabbit), NR-13807. Horseradish peroxidase (HRP)-conjugated goat anti-mouse immunoglobulin secondary antibody (Bio-Rad) was utilized at 1:5,000 for detection of RpoB, EsxA, and FLAG. HRP-conjugated goat anti-rabbit IgG secondary antibody (Bio-Rad) was utilized at 1:5,000 for detection of EsxB and Mpt32. All proteins were detected using the LumiGLO Chemiluminescent Substrate Kit (SeraCare, Milford, MA) and X-ray film (RPI, Mt. Prospect, IL).

Preparation of Lipids and Thin-Layer Chromatography

M. marinum cultures were grown to mid-log phase in 50 mL of 7H9 supplemented with 0.1% Tween-80. Bacteria were harvested by centrifugation and washed 3x in filter-sterilized PBS. Washed pellets were resuspended in 1.6 mL of methanol (VWR), before being transferred to a borosilicate glass vial (VWR) containing 3.6 mL of $CHCl_3$ (Electron Microscopy Sciences). These were gently agitated and left at room temperature overnight. The lipid extraction was filtered through #1 Whatman filter paper (GE) using a Buchner funnel and the filtrate was transferred to fresh vial containing 1 mL of ddH2O. These were agitated vigorously on a vortex, before the phases were allowed to settle for 3 hours. The aqueous phase was removed using a Pasteur pipette, and the organic phase was transferred to a new borosilicate vial. 500 µL of this organic phase was transferred to a new borosilicate vial and dried to a powder by standing uncapped overnight in a chemical fume hood at room temperature. Each dried lipid pellet was dissolved in a 1:2 methanol:$CHCl_3$ solution. ~6 µL of each clarified lipid was spotted using a Pasteur pipette on a silica gel 60 F_{254} TLC plate (EMD) before being run for ~30 minutes in a TLC jar using a 50:1 petroleum ether :ethyl acetate solution. TLC plates were stained for one minute in phosphomolybdic acid (Sigma) and developed by heating with a heat gun followed by immediately imaging using a flatbed scanner. 1.5 µL of H37Rv, Purified Phthiocerol Dimycocerosate (PDIM) , which was obtained through BEI Resources, NIAID, NIH: *Mycobacterium tuberculosis*, Strain H37Rv, Purified Phthiocerol Dimycocerosate (PDIM), NR-20328, was used as a positive control. Retention factors for each TLC plate were calculated exactly as described previously (E.A. Williams et al. Infect Immun., 85(2). pii: e00653-16, 2017. doi: 10.1128/IAI.00653-16).
