# Supplementary material for: EspM Is a Conserved Transcription Factor That Regulates Gene Expression in Response to the ESX-1 System
Source: mBio. 2020 Feb 4;11(1):e02807-19. doi: 10.1128/mBio.02807-19 (PMC7002343; doi:10.1128/mBio.02807-19)
Supplement: FIG S3 [file mBio.02807-19-sf003.pdf]

Figure S3

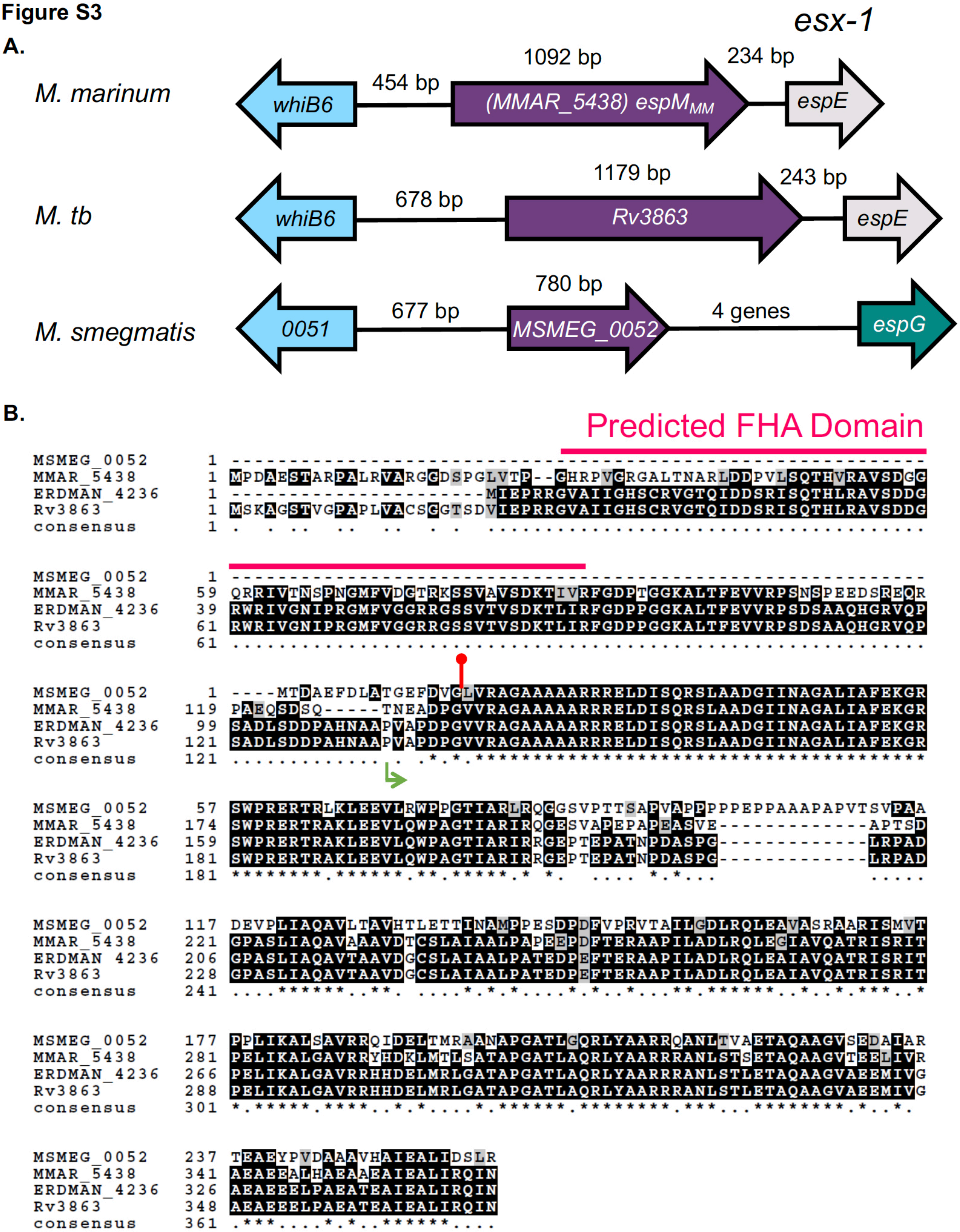
esx-1
A.
M. marinum
whiB6
454 bp
1092 bp
(MMAR_5438) espM_MM
234 bp
espE
M. tb
whiB6
678 bp
1179 bp
Rv3863
243 bp
espE
M. smegmatis
0051
677 bp
780 bp
MSMEG_0052
4 genes
espG
B.
Predicted FHA Domain
MSMEG_0052 1 ---
MMAR_5438 1 MPDAESTARPALRVARGGDSPGLVTP--GHRPVGRGALTNARLDDPVLSQTHVRAVSDGG
ERDMAN_4236 1 ----------------------MIEPRRGVAIIGHSCRVGTQIDDSRISQTHLRAVSDDG
Rv3863 1 MSKAGSTVGPAPLVACSGGTSDVIEPRRGVAIIGHSCRVGTQIDDSRISQTHLRAVSDDG
consensus 1
MSMEG_0052 1 ---
MMAR_5438 59 QRRIVTNSPNGMFVDGTRKSSVAVSDKTIVRFGDPTGGKALTFEVVRPSNSPEEDSREQR
ERDMAN_4236 39 RWRIVGNIPRGMFVGGRRGSSVTVSDKTLIRFGDPPGGKALTFEVVRPSDSAAQHGRVQP
Rv3863 61 RWRIVGNIPRGMFVGGRRGSSVTVSDKTLIRFGDPPGGKALTFEVVRPSDSAAQHGRVQP
consensus 61
MSMEG_0052 1 ----MTDAEFDLATGEFDVGLVRAGAAAAARRRELDISQRSLAADGIINAGALIAFEKGR
MMAR_5438 119 PAEQSDSQ-----TNEADPGVVRAGAAAAARRRELDISQRSLAADGIINAGALIAFEKGR
ERDMAN_4236 99 SADLSDDPAHNAAPVAPDPGVVRAGAAAAARRRELDISQRSLAADGIINAGALIAFEKGR
Rv3863 121 SADLSDDPAHNAAPVAPDPGVVRAGAAAAARRRELDISQRSLAADGIINAGALIAFEKGR
consensus 121
MSMEG_0052 57 SWPRERTRLKLEEVLRWPPGTIARLRQGGSVPTTSAPVAPPPPPEPPAAAPAPVTSVPAA
MMAR_5438 174 SWPRERTRAKLEEVLQWPAGTIARIRQGESVAPEPAPEASVE------------APTSD
ERDMAN_4236 159 SWPRERTRAKLEEVLQWPAGTIARIRRGEPTEPATNPDASPG------------LRPAD
Rv3863 181 SWPRERTRAKLEEVLQWPAGTIARIRRGEPTEPATNPDASPG------------LRPAD
consensus 181
MSMEG_0052 117 DEVPLIAQAVLTAVHTLETTINAMPPESDPDFVPRVTAILGDLRQLEAVASRAARISMVT
MMAR_5438 221 GPASLIAQAVAAAVDTCSLAIAALPAPEEPDFTERAAPILADLRQLEGIAVQATRISRIT
ERDMAN_4236 206 GPASLIAQAVTAAVDGCSLAIAALPATEDPEFTERAAPILADLRQLEAIAVQATRISRIT
Rv3863 228 GPASLIAQAVTAAVDGCSLAIAALPATEDPEFTERAAPILADLRQLEAIAVQATRISRIT
consensus 241
MSMEG_0052 177 PPLIKALSAVRRQIDELTMRAANAPGATLGQRLYAARRQANLTVAETAQAAGVSEDAIAR
MMAR_5438 281 PELIKALGAVRRYHDKLMTLSATAPGATLAQRLYAARRRANLSTSETAQAAGVTEELIVR
ERDMAN_4236 266 PELIKALGAVRRHHDELMRLGATAPGATLAQRLYAARRRANLSTLETAQAAGVAEEMIVG
Rv3863 288 PELIKALGAVRRHHDELMRLGATAPGATLAQRLYAARRRANLSTLETAQAAGVAEEMIVG
consensus 301
MSMEG_0052 237 TEAEYPVDAAAVHAIEALIDSLR
MMAR_5438 341 AEAEEALHAEAAEAIEALIRQIN
ERDMAN_4236 326 AEAEEELPAEATEAIEALIRQIN
Rv3863 348 AEAEEELPAEATEAIEALIRQIN
consensus 361
