# Supplementary material for: EspM Is a Conserved Transcription Factor That Regulates Gene Expression in Response to the ESX-1 System
Source: mBio. 2020 Feb 4;11(1):e02807-19. doi: 10.1128/mBio.02807-19 (PMC7002343; doi:10.1128/mBio.02807-19)
Supplement: TABLE S2 [file mBio.02807-19-st002.pdf]

Table S2: *M. marinum* strains, plasmids and primers used in this study.

**Strains**		
**Name**	**Genotype**	**Reference**
M. marinum M strain	Wild type; parent for all strains	ATCC BAA-535
Δ*whiB6*	M with a deletion in the *whiB6* (*MMAR_5437*) gene	R. E. Bosserman *et al. Proc Natl Acad Sci U S A* 114(50):E10772-E10781, 2017 doi: 10.1073/pnas.1710167114.
Δ*whiB6*::*lacZ*+	M with the *whiB6* gene replaced with the *lacZ* gene	This study
Δ*whiB6*::*lacZ*+Δ *eccCb*$_1$	Δ*whiB6*::*lacZ*+ strain with a deletion of the *eccCb*$_1$ (*MMAR_5446*) gene.	This study
whiB6Fl	M with *whiB6* allele tagged with 3X-FLAG at C-terminus, at the *whiB6* locus; parental strain for the following strains	R. E. Bosserman *et al. Proc Natl Acad Sci U S A* 114(50):E10772-E10781, 2017 doi: 10.1073/pnas.1710167114.
Δ*whiB6*Δ*espM*	M with deletions in the *whiB6* (*MMAR_5437*) and *espM* (*MMAR_5438*) genes.	This study
*whiB6Fl*Δ*espM*	*whiB6Fl* with deletion of the *espM* (*MMAR_5438*) gene	This study
*whiB6Fl*Δ*eccA*$_1$	*whiB6Fl* with deletion of the *eccA*$_1$ (*MMAR_5443*) gene	This study
*whiB6Fl*Δ*espM*Δ *eccA*$_1$	*whiB6Fl*Δ*espM* with deletion of the *eccA*$_1$ (*MMAR_5443*) gene	This study
*whiB6Fl*Δ*eccB*$_1$	*whiB6Fl* with deletion of the *eccB*$_1$ (*MMAR_5444*) gene	R. E. Bosserman *et al. Proc Natl Acad Sci U S A* 114(50):E10772-E10781, 2017 doi: 10.1073/pnas.1710167114.
*whiB6Fl*Δ*espM*Δ *eccB*$_1$	*whiB6Fl*Δ*espM* with deletion of the *eccB*$_1$ (*MMAR_5444*) gene	This study
*whiB6Fl*Δ*eccCa*$_1$	*whiB6Fl* with deletion of the *eccCa*$_1$ (*MMAR_5445*) gene	This study
*whiB6Fl*Δ*espM*Δ *eccCa*$_1$	*whiB6Fl*Δ*espM* with deletion of the *eccCa*$_1$ (*MMAR_5445*) gene	This study

*whiB6Fl*Δ*eccCb*$_1$	*whiB6FL* with deletion of deletion of the *eccCb*$_1$ (*MMAR_5446*) gene	R. E. Bosserman *et al. Proc Natl Acad Sci U S A* 114(50):E10772-E10781, 2017 doi: 10.1073/pnas.1710167114.
*whiB6Fl*Δ*espM*Δ *eccCb*$_1$	*whiB6Fl*Δ*espM* with deletion of the *eccCb*$_1$ (MMAR_5446) gene	This study
*whiB6Fl*Δ*eccD*$_1$	*whiB6Fl* with deletion of the *eccD*$_1$ (MMAR_5452) gene	R. E. Bosserman *et al. Proc Natl Acad Sci U S A* 114(50):E10772-E10781, 2017 doi: 10.1073/pnas.1710167114.
*whiB6Fl*Δ*espM*Δ *eccD*$_1$	*whiB6Fl*Δ*espM* with deletion of the *eccD*$_1$ (MMAR_5452) gene	This study
*whiB6Fl*Δ*eccE*$_1$	*whiB6Fl* with deletion of the *eccE*$_1$ (MMAR_5458) gene	R. E. Bosserman *et al. Proc Natl Acad Sci U S A* 114(50):E10772-E10781, 2017 doi: 10.1073/pnas.1710167114.
*whiB6Fl*Δ*espM*Δ *eccE*$_1$	*whiB6Fl*Δ*espM* with deletion of the *eccE*$_1$ (MMAR_5458) gene	This study
**Plasmids**		
**Name**	**Genotype/ Phenotype**	**Reference**
p2NIL	kan^R, amp^R, *oriE; Parental vector for allelic exchange*	T. Parish and N.G.Stoker, Microbiology, 146 (Pt 8):1969-75,2000, https://doi.org/10.1099/00221287-146-8-1969
pGOAL19	*amp*^{R}, GOAL cassette includes *hyg*^{R}, *lacZ, sacB, oriE; Parental vector for allelic exchange*	T. Parish and N.G.Stoker, Microbiology, 146 (Pt 8):1969-75,2000, https://doi.org/10.1099/00221287-146-8-1969
p2NILΔ*espM*GOAL	*espM*$_{MM}$ flanking regions, *kan*^{R}, *hyg*^{R}, *sacB*, *lacZ*	This study
p2NILΔ*eccA*$_1$GOAL	*eccA*$_1$ flanking regions, *kan*^{R}, *hyg*^{R}, *sacB*, *lacZ*	This study
p2NILΔ*eccB*$_1$GOAL	*eccB*$_1$ flanking regions, *kan*^{R}, *hyg*^{R}, *sacB*, *lacZ*	R. E. Bosserman *et al. Proc Natl Acad Sci U S A* 114(50):E10772-E10781, 2017

		doi: 10.1073/pnas.1710167114.
p2NILΔ*eccCa₁*GOAL	*eccCa₁* flanking regions, *kan*^{R}, *hyg*^{R}, *sacB*, *lacZ*	R. E. Bosserman *et al. Proc Natl Acad Sci U S A* 114(50):E10772-E10781, 2017 doi: 10.1073/pnas.1710167114.
p2NILΔ*eccCb₁*GOAL	*eccCb₁* flanking regions, *kan*^{R}, *hyg*^{R}, *sacB*, *lacZ*	E.A. Williams et al. Infect Immun., 85(2). pii: e00653-16, 2017. doi: 10.1128/IAI.00653-16
p2NILΔ*eccD₁* GOAL	*eccD₁* flanking regions, *kan*^{R}, *hyg*^{R}, *sacB*, *lacZ*	R. E. Bosserman *et al. Proc Natl Acad Sci U S A* 114(50):E10772-E10781, 2017 doi: 10.1073/pnas.1710167114.
p2NILΔ*eccE₁* GOAL	*eccE₁* flanking regions, *kan*^{R}, *hyg*^{R}, *sacB*, *lacZ*	R. E. Bosserman *et al. Proc Natl Acad Sci U S A* 114(50):E10772-E10781, 2017 doi: 10.1073/pnas.1710167114.
p2NILΔ*whiB6* GOAL	*whiB6* flanking regions, *kan*^{R}, *hyg*^{R}, *sacB*, *lacZ*	R. E. Bosserman *et al. Proc Natl Acad Sci U S A* 114(50):E10772-E10781, 2017 doi: 10.1073/pnas.1710167114.
p2NILΔ*whiB6*Δ*espM* GOAL	*whiB6* and *espM* flanking regions, *kan*^{R}, *hyg*^{R}, *sacB*, *lacZ*	This study
p2NIL*whiB6::lacZ*GOAL	*lacZ* with *whiB6* flanking regions, *kan*^{R}, *hyg*^{R}, *sacB*, *lacZ; used to generate a transcriptional reporter at the whiB6 locus.*	This study
pMH406Hyg	*M. tuberculosis esxBA* behind mycobacterial optimal promoter (MOP), *oriE*, L5 integrase, *hyg*^{R}	Original plasmid from (K.M. Guinn et al, Mol Microbiol 51(2):359-70, 2004. https://doi.org/10.1046/j.1365-2958.2003.03844.x); hyg^R derivative was a gift from the Jeffery S. Cox laboratory
pMV306Hyg	Promoterless shuttle vector, *oriE*, L5 integrase, *hyg*^{R}	C. K. Stover et al, Dev Biol Stand (**82**): 163-170, 1994
p$_{MOP}$*lacZ*	*lacZ* behind the pMOP promoter, *hyg*^{R}	This study
p$_{MOP}$*eccA₁*	*eccA₁* from *M. marinum* behind MOP promoter, L5	This study

	integrase, *hygR*.	
pR2Hyg-dsRed	*dsRed* expressed behind the *msp12* promoter	C.L. Cosma, O. Humbert, and L. Ramakrishnan, Nat Immunol 5: 828-835, 2004.DOI: 10.1038/ni1091
p_{MOP}mOrange	mOrange (from Addgene #29770) behind mycobacterial optimal promoter (MOP), *oriE*, L5 integrase, hyg^R	This study
p_{msp}mOrange	mOrange behind the *msp* promoter, *oriE*, L5 integrase, hyg^R	This study
$p_{msp}espM_{Mm}$	*espM* (*MMAR_5438*) behind *msp* promoter, L5 integrase, hyg^R	This study
$p_{msp}espM_{Mt}$	*espM* (*ERDMAN_4236*) from *M. tuberculosis* str. Erdman behind *msp* promoter, L5 integrase, hyg^R	This study
$p_{msp}espM_{Ms}$	*espM* (*MSMEG_0052*) from *M. smegmatis* str. mc^2155 behind *msp* promoter, L5 integrase, hyg^R	This study
pKT25	Bacterial 2-hybrid prey vector. T25 fragment of *Bordatella pertussis cyaA* gene, P15A origin, kan^R	Euromedex
pKT25*espMMt*	*espM* (*ERDMAN_4236*) from *M. tuberculosis* str. Erdman fused to T25 fragment of *cyaA, kan*^{R}	This study.
pET15b	T7 expression vector with N-terminal 6xHis tag, amp^R	Novagen
pET15*mCherry*	*mCherry* (from Addgene #29769) flanked by NdeI and SpeI restriction sites in the pET15 vector, amp^R	This study
pET15$espM_{Mm}$	$espM_{MM}$ with an N-terminal His6 affinity tag, amp^R	This study
pET15$espM_{Mm}$NT	N-terminal half of $espM_{MM}$ (aa 1-133) with N-terminal His6 affinity tag, amp^R	This study
pET15$espM_{Mm}$CT	C-terminal half of $espM_{MM}$ (aa 127-363) with N-terminal His6 affinity tag, amp^R	This study.
pET15$espM_{Ms}$full	Heterologous expression of full-length *M. smegmatis* espM ORF with N-terminal His6 affinity tag. amp^R	This study.
pET15$espM_{Mt}$	$espM_{Mt}$ with an N-terminal His6 affinity tag, amp^R	This study.
pET29	T7 expression vector with C-terminal 6xHis tag, kan^R	Novagen
pET29*whiB6*	$whiB6_{MM}$ with an C-terminal His6 affinity tag, kan^R	This study.

pMV306*whiB6::lacZ*+	Upstream sequence of *whiB6* fused to *lacZ.* L5 integrase, hyg^R	This study.
pMV306*espM::lacZ*+	Upstream sequence of *espM* fused to *lacZ.* L5 integrase, hyg^R	This study.
**Primers**		
**Name**	**Sequence (5'→ 3')**	**Application**
orb14	TCAATAGCCTCGGCGGCTTC	Amplification of 1.5 kb upstream of *whiB6,* R. E. Bosserman *et al. Proc Natl Acad Sci U S A* 114(50):E10772-E10781, 2017 doi: 10.1073/pnas.1710167114.
orb72	ATCCACGACTGCCCGAAAC	Used for verification of *eccCb*$_1$ deletion.
orb73	CGAGCGTTGCCGCTCAATAG	
ors221	TGATGCCCGGCCGCGTCC	Amplification of sequence downstream of *whiB6*, R. E. Bosserman *et al. Proc Natl Acad Sci U S A* 114(50):E10772-E10781, 2017 doi: 10.1073/pnas.1710167114.
OKS18	/BiotinTEG/AAACGCCCAGCGGAATCT	Amplification of 979 bp fragment containing the *whiB6* promoter used as ~1 kb probe in promoter pulldown assays.
OKS19	/BiotinTEG/TTGATGATCCCGTCGGCT	
RpoA Fwd	/BiotinTEG/ATGCTGATCTCTCAGCGGC	Amplification of 1044 bp fragment containing the *whiB6* promoter used as ~1 kb probe in promoter pulldown and EMSA assays.
RpoA Rev	/BiotinTEG/TTAGAGCTGTTCGGTTTCGGC	
OKS77	GATAACACGGGATCATCCAG	Amplification of 555 bp fragment containing the *whiB6* promoter used as ~500 bp probe in EMSA assays.
OKS88	GTTGCAGGTTCGTAACCTC	
OKS94	ATGGAATCGACCGGGATGC	Amplification of 499 bp fragment of *rpoA* gene used as ~500 bp probe in EMSA assays.
5438FqRT	CGTCACCAACAGCCCAAACG	

5438RqRT	CTGCGCTGACTGATGTCGAG	Primers for qRT-PCR expression of the interior of *espM*.
SigA F	TCGAGGTGATCAACAAGCTG	Primers for qRT-PCR expression of the interior of *sigA*.
SigA R	TGGATCTCCAGCACCTTCTC	
ORS225	AGATTCCGCTGGGCGTTTGC	Primers for qRT-PCR expression of the interior of *whiB6*.
ORS226	TCTGCCAGCGACCGAAGTTG	
OKS109	CCTCTGCCTGCCTAGC	Amplification of 515 bp fragment of *whiB6* Erdman promoter used as ~500 bp probe in EMSA assays.
OKS110	CTCGCAGATGTGTCTGAGAG	
OMF004	GAGCCACCCGCAGTTCGAAAAATGACTAGTCGGGACCGCTCAGGCGT	Amplify pMOP for mOrange cloning
OMF042	CATGCTGGACTCCTGAATTCTGCAGCT	
OMF047	GAATTCAGGAGTCCAGCATGGTGAGCAAGGGCGAGGAG	Clone mOrange into pMOP
OMF048	CGAACTGCGGGTGGCTCCAATTTCCCTTGTACAGCTCGTCCATGCC	
OMF049	GTTGGACTCAAGACGATAGTTACCGGATAAG	Plasmid amplification from pBR322 origin
OMF050	CTTATCCGGTAACTATCGTCTTGAGTCCAAC	
OMF072	GATCTTTAAATCTAGAGGATCTGACCCGCTCCACAAC	Amplification of MSP promoter
OMF074	TCTAGATTTAAAGATCTGGTACCGCGG	Amplification of pMV306 vector; MSP promoter swap
OMF082	CATATGTATATCTCCTTCTTAAATCTAGATTTAAAGATCTGGTACCGG	Amplification of MSP promoter
OMF083	CAGATCTTTAAATCTAGATTTAAGAAGGAGATATACATATGGTGAGCAAGGGCGAGGAG	Amplification of pMOP-mOrange for MSP promoter swap.
OMF096	ACGAGCGTGACACCACGATGCC	Amplification of p2NIL vector
OMF097	ACGGTGCCTGACTGCGTTAGCAATTTAACTG	

OMF173	GCTGATTAAATATGATGAAAACGGCAACC	Amplification from *lacZ*, for confirmation of reporter location and orientation
OMF174	GGTTGCCGTTTTCATCATATTTAATCAGC	
OMF180	CGTGGTGTCACGCTCGTTGGCCAACAATCTGATCTCGG	Amplification of upstream region flanking the *eccA*$_1$ gene; Used for construction of p2NILΔ*eccA*$_1$ GOAL suicide plasmid
OMF181	CATATTGAGGTGCTTAAGTGCACTTTCGAACAAACCGGC	
OMF182	CGAAAGTGCACTTAAGCACCTCAATATGAGAGAGTGAAACATGG	Amplification of downstream region flanking the *eccA*$_1$ gene; Used for construction of p2NILΔ*eccA*$_1$ GOAL suicide plasmid
OMF183	ACGCAGTCAGGCACCGTGTAGGGCAGGATCTTCTGCACG	
OMF184	CTGCGAACATGTCGGAATCC	Used for verification of *eccA*$_1$ deletion
OMF185	GCCACCAAGTTTTCCAGCTGG	
OMF190	ACTCCTGCAGGCAGCCAAAACG	Used for verification of *eccB*$_1$ deletion
OMF191	GGCATCATCAGCATGTATGGCG	
OMF196	GGAAATTTGTTCAGTTGCAGTCACC	Used for verification of *eccCa*$_1$ deletion
OMF197	AGGTCGATGCAATAGAACTGGACC	
OMF202	GTGTCGGAACAGCAGTTGGTCC	Used for verification of *eccD*$_1$ deletion
OMF203	CTCCAGTTGCTGCTATCGGCG	
OMF208	GGTGGTCAATGCTGTCGCGG	Used for verification of *eccE*$_1$ deletion
OMF209	CTGTTCAAGATTTCCTGCTGATCCACC	
OMF313	CATATGTATATCTCCTTCTTAAAGTTAAACAAAATTATTTCTAGAGG	Amplification of pET29 vector.
OMF351	ACGTCGTACGGGTACATATCTAAGCGTTCCTCCATAAAGCAAAAGCGTTGC	Amplification of sequence immediately upstream of *whiB6* gene to introduce linker for HA epitope and fusion to *lacZ*.
OMF352	ACGTCGTACGGGTACATATCTAAGCGTTCCTCCATAAAGCAAAAGCGTTGC	Reverse primer for amplification of *lacZ* with linker for sequence downstream of *whiB6*.

OMF353	ATGTACCCGTACGACGTGCCCGACTACGCGGATCCCGTCGTTTTACAACG	Forward primer for amplification of *lacZ* with linker encoding HA epitope for fusion to sequence immediately before *whiB6*.
OMF355	GCGGTACCAGATCTTTAGTACGTCGATTCCTCGCTCGCGGC	Amplification of *whiB6* and flanking sequence for cloning into pMV306Hyg.
OMF356	ATCGTACGCTAGTTAACGGCTTCTGGTCCGCTGGATGCTCG	
OMF512	ACTAGTAACTAGCATAACCCCTTGGGGC	Amplification of pET15 vector
OMF513	CATATGATGATGATGATGATGGCTGC	
OMF514	ATCATCATCATCATATGAGCAAGGGCGAGGAGGAT	Clone mCherry into pET15 wit flanking NdeI and SpeI restriction sites.
OMF515	TTATGCTAGTTACTAGTCTACTTGTACAGCTCGTCCATGCC	
OMF606	CACCACCACCACCACCACTG	Amplification of pET29 vector.
OMF607	AGGAGATATACATATGACTGCAACTGCTCTGTACGAGATTCCG	Amplification of *whiB6* for cloning into pET29.
OMF608	GGTGGTGGTGGTGGTGTGCCGATTGGGCGGTGATCC	
OMF615	GTGGTGTCACGCTCGTCCGGTTGAGCAGCAGGATCGATCCC	Amplification of upstream region flanking the *espM* gene. Used for construction of p2NILΔ*espM* GOAL suicide plasmid.
OMF616	CCTCGGCCTTAAGCGATTCGGCGTCGGGCATGTACG	
OMF617	CGAATCGCTTAAGGCCGAGGCTATTGAAGCGCTCATCC	Amplification of downstream region flanking the *espM* gene. Used for construction of p2NILΔ*espM* GOAL suicide plasmid.
OMF618	CGCAGTCAGGCACCGTGGTTTGGCTCGCTATGGCTTTGGTAGG	
OMF619	GCTGGATGTCGTTCAAGAAACGTAGCG	Used for verification of *espM* deletion
OMF620	GTGGTCGACGCACGAATTTCTTGG	
OMF621	TGGCAGTCGACGCCGATACC	Used for verification of p2NILΔ*espM*GOAL suicide plasmid integration (merodiploid).
OMF622	CATGGGGGTAGACCTTTCCTCTACG	
OMF624	TCATCATCATCATATGCCCGACGCCGAATCGACTG	

OMF625	TATGCTAGTTACTAGTTCAATTGATTTGACGGATGAGCGC	Amplification of $espM_{MM}$ for cloning into pET15
OMF626	ATGCTAGTTACTAGTTCAACCAGGATCGGCTTCGTTGGTCTGAG	Amplification of N-terminal half of $espM_{MM}$ ORF for cloning into pET15
OMF627	CATCATCATCATATGACCAACGAAGCCGATCCTGGTG	Amplification of C-terminal half of $espM_{MM}$ ORF for cloning into pET15
OMF630	ACTAGTCGGGACCGCTCAGGCGTCC	Amplification of pMOP vector.
OMF631	TCATCATCATCATATGACCGACGCGGAGTTCGACC	Amplification of *M. smegmatis espM* for cloning into pET15 expression vector.
OMF632	ATGCTAGTTACTAGTTCAGCGCAGCGAGTCGATGAGC	Amplification of *M. smegmatis espM* for cloning into pET15 expression vector.
OMF645	GGAGTCCAGCCATATGACTGATCGCCTGGCCGG	Amplification of $eccA_1$ ORF for cloning into pMOP.
OMF646	AGCGGTCCCGACTAGTCACTCTCTCATATTGAGGTGTGCATGCAC	
OMF701	GATCTTTAAATCTAGAGACACAGCGTCTTGGCCTCCTCG	Amplify upstream of *espM* ORF for fusion to *lacZ* and cloning into pMV306Hyg.
OMF709	CCAAGCCTTAAGACCGAAAACGTAGCGTCTGAGACCTAGG	Used for verification of *whiB6 espM* double deletion.
OMF733	GTCGTACGGGTACATGTACGTCGATTCCTCGCTCGCGGC	Amplify upstream of *espM* ORF for fusion to *lacZ* and cloning into pMV306Hyg.
OMF734	TCGTACGCTAGTTAACTCATTTTTGACACCAGACCAACTGG	Amplify *lacZ* with linker for cloning into pMV306Hyg.
RpoA-Fwd	ATGCTGATCTCTCAGCGGC	Amplification of *M. marinum rpoA* ORF
RpoA-Rev	TTAGAGCTGTTCGGTTTCGGC	
V-KT25-F	ACTAGTGTCGACTCTAGAGGATCCCCGGGTACCTAAGTAACTAAGAATTCGGCCG	Amplification of pKT25 vector.
V-KT25-R	CATATGTCTAGAGAATTCAGCCCGCCGCGTGCGCGCCAGGTAATCG	

ERD-4236-F	GAATTCTCTAGACATATGATTGAGCCCCGTCGCGG	Amplify *ERDMAN_4236* with linkers for cloning into pKT25.
ERD-4236-R	CTCTAGAGTCGACACTAGTTCAATTGATCTGACGGATCAGGGC	
